# Digital marketplaces in European research landscape: A systematic literature review

**DOI:** 10.12688/openreseurope.18657.1

**Published:** 2024-10-17

**Authors:** Georgios Nikoletos, Iordanis Papoutsoglou, Georgios Spanos, Alexandros Nizamis, Antonios Lalas, Konstantinos Votis, Dimitrios Tzovaras

**Affiliations:** 1 Information Technologies Institute, Centre for Research and Technology-Hellas, Thessaloniki, Makedonia Thraki, Greece

**Keywords:** Marketplace. Software Technologies, Blockchain, Payment Mechanisms

## Abstract

The e-commerce and digital technologies growth, has led to the emergence of various electronic marketplaces having the ability to connect parties across geographical locations, thus offering convenience and flexibility. The European Union recognizes the prowess of digital marketplaces and for this reason, many EU-funded projects presented e-marketplaces in various sectors. For this reason, a Systematic Literature Review (SLR) is proposed to summarize recent studies in the field, providing a comprehensive overview of specific business and technical characteristics, and extracting valuable insights. From the SLR, 26 primary studies have been extracted during 2013–2023. The analysis highlighted that there are five marketplace types in terms of market offerings, catering to multiple sectors of economy. Moreover, the emergence of the blockchain technology has led to the development of decentralized marketplaces, offering greater security, and transparency. This trend is also reflected by the results alongside with some useful outcomes regarding implementation technologies, interoperability and deployment. Finally, the results highlighted that the exploitation of these marketplace is an open issue.

## 1. Introduction

A digital marketplace refers to an online software platform that brings together buyers and sellers to facilitate the exchange of goods and services ( Jiang & Balasubramanian, 2014). Over the past years, the popularity and use of digital marketplaces have exploded ( Costa Melo *et al.*, 2022), driven by the exponential rise of e-commerce and e-procurement solutions; and by the use of different types of technological and innovative solutions in everyday life ( Shirzad & Bell, 2013). Through this technological outburst, digital marketplaces are now being used across many industries and sectors of the economy; since they offer a number of advantages in comparison to traditional brick-and-mortar stores and other forms of e-commerce. Undoubtedly, the ease of business globalization through digital marketplaces provides a wider range of products to potential customers, thus increasing product visibility and accessibility; while also making it easy for customers to compare prices from multiple vendors ( Cano *et al.*, 2022). According to the aforementioned, it is obvious that commerce through the Internet has enhanced the bargaining power of the customer’s side ( Laudon & Laudon, 2016).

In addition to these benefits, digital marketplaces also offer a number of advanced features, such as personalized recommendations, real-time pricing, availability information, integrated payment and shipping solutions ( Shirzad & Bell, 2013), which in turn make the shopping experience faster, easier, and more convenient. However, the expansion of digital marketplaces has also introduced novel barriers, including increased competition, sophisticated cybersecurity requirements, and the necessity to handle customer trust and satisfaction ( Zahedi *et al.*, 2010). To address these hurdles, businesses in the digital marketplace ecosystem are investing in new technologies such as artificial intelligence, machine learning, and blockchain, having as major goals the customer experience improvement, fraudulent activities mitigation, and ubiquitous operations.

Typical electronic marketplaces can be described as web applications that allow commercial transactions from different sources in a convenient way ( Wang *et al.*, 2013). However, the ever increasing number of technological advancements in the wider area of Information Technology ( Buyya *et al.*, 2009) and in the global environment of multi-stakeholders and services allow digital marketplaces to act as a focal point for the provision of heterogeneous “products”. In other approaches ( Nizamis *et al.*, 2018), marketplaces can be considered as ecosystems that virtual agents represent providers and consumers, to automate the decision making and exchange of goods or services. Due to the above, different types of marketplaces have now emerged (except from the traditional e-commerce platforms where physical products are traded), with software, data, and services marketplaces being amongst the most popular. In addition, multi-type marketplaces are becoming more and more common, where different types of “products” are being traded among interested parties and stakeholders.

Preliminary research in the aforementioned emerging field has pointed out that digital marketplaces are being developed across different industries, such as manufacturing, healthcare, automotive, cloud computing, cybersecurity, and many more ( Hänninen & Smedlund, 2021). These online marketplaces differ from traditional e-commerce platforms since their main objective is to design, develop, and maintain a sustainable centralized or decentralized web-based platform that can serve as an access point for online trading, buying, and/or using of various tangible or intangible ”products” in a proprietary manner ( Atluri *et al.*, 2017).

By further expanding the preliminary research, it has shown that a significant number of European projects (either funded directly from the European Commission or through grants from Programmes for Research and Technological Development, i.e. HORIZON2020 ^1^, Horizon Europe ^2^, FP7 ^3^ 3, etc.) focus on developing digital marketplaces in order to create an ecosystem that can assist to exploitation activities since they can act as a one-stop-place where various software services, data, tools, applications, and products, are being shared and used from the different beneficiaries, associated partners and/or external entities. For these reasons, the EU has encouraged the use of digital technologies to enhance competitiveness and innovation in the region, and digital marketplaces have been a key part of this effort. It is also worth-mentioning that a main differentiation of the digital marketplaces from EU-funded projects in comparison with other marketplaces presented in the literature is that can also serve a higher community purpose, such as public health and security apart from exploitation purposes and is prominent to investigate if there are specific characteristics either business or technical toward this direction. Furthermore, since the nature of the EU-funded projects is research oriented, it would be interesting to explore the different research areas covered by such marketplaces. Additionally, considering the huge amount of the total budget invested from the EU in different EU funding programs (e.g. the total budget of the Horizon 2020 program was nearly 80 billion ^4^), in which a large number of projects contains digital marketplaces, it would be valuable to assess the research footprint of these digital marketplaces. From all the above, it is obvious that there is a strong research interest for investigation of the EU-funded digital marketplaces.

Considering all the aforementioned, the purpose of the present research work is to streamline and extract aggregated knowledge from recent studies that present digital marketplaces that were developed in the course of EU-funded projects. Although several digital marketplaces have been developed and deployed in many European projects, they have not yet drawn much attention from the research community. To that end, the key objectives of this work are: (a) to provide a detailed overview of either fully developed or just prototypes of digital marketplaces delivered from European funding; (b) to classify them based on specific characteristics; (c) to identify research gaps and recommend areas for future study; and (d) to steer and motivate future research to foster innovation regarding several aspects in the field. For these reasons, a Systematic Literature Review (SLR) is presented to incorporate the aforementioned objectives as it sets out to search, compile, highlight, assess, and categorize primary studies in the field in a rigidly precise, typical, and systematic way.

The structure of the rest of the work is the following. Section 2 describes the related work and similar SLRs in the area of digital marketplaces, while Section 3 presents the methodology that this paper is based upon, by providing insight into the main research questions that are to be addressed through this work, thus identifying the need for this research. Moreover, Section 4 further examines the research questions that need to be addressed and showcases the general and the more specific results that were deducted during this research. Finally, Section 5 provides a comprehensive discussion of the research obstacles identified in this review and suggests potential avenues for future research, while Section 6 summarizes the conclusions of this review paper.

## 2. Related work

Digital marketplaces have gained significant academic and practical interest, reflecting a dynamic meeting point of technological innovation and economic activity. This section investigates existing literature, emphasizing the evolution of digital marketplaces, methodologies employed in prior reviews, key findings, and identified research gaps.

### 2.1. Business-oriented reviews

Studies like Shirzad and Bell (2013) and Cano *et al.* (2022) have adopted systematic literature reviews and bibliometric analyses, focusing on the impact of digital platforms across industries. These reviews offer an analytical view on how digital platforms facilitate economic activities, with a focus on sustainable practices. Similar studies like Täuscher and Laudien (2018) deal with the classification and understanding of platform business models within the context of digital marketplaces, while Shree *et al.*, 2021 offer a systematic review that contains a research on digital platforms in B2B contexts, supported by the Technology-Organization-Environment (TOE) framework and the Diffusion of Innovation (DOI) theory.

Shirzad and Bell (2013) emphasize the importance of the TOES framework in understanding e-procurement marketplace flexibility, suggesting a mixed-methods approach combining quantitative and qualitative analyses. This perspective is helpful in understanding the challenges and opportunities inherent in the deployment and management of e-procurement systems. By categorizing flexibility into visible domains, the authors provide a framework for analyzing e-procurement marketplaces, offering valuable insights into how these platforms can better accommodate the changing demands of the digital economy.

Cano *et al.* (2022) examine the mutual dependency between e-marketplaces and open innovation, leveraging a decade-long bibliometric review to highlight sustainability's pivotal role. Their research unveils the developing focus on the relationship between e-marketplaces and open innovation mechanisms, underscoring the essential role of sustainability in shaping future marketplace models. By analyzing trends across varying timeframes, the study not only maps out the evolution of academic discourse but also identifies important themes such as the integration of environmental, social, and economic dimensions of sustainability. This analysis clarifies the critical pathways through which e-marketplaces can leverage open innovation to foster sustainable development, thus providing a robust framework for future research endeavors in the meeting point of digital commerce, innovation, and sustainability.

The research of Täuscher and Laudien (2018) meticulously defines six distinct types of marketplace business models, emphasizing the varied mechanisms through which these platforms create, deliver, and capture value. By leveraging a robust analytical framework, the study not only sheds light on the operational complexities and strategic necessities of platforms like Airbnb and Uber but also presents the broader implications for innovation, competition, and regulation within digital ecosystems. Furthermore, their work contributes to the ongoing discourse on the sharing economy and gig economy by providing an understanding of how digital platforms can foster novel forms of economic interaction and value exchange, hence challenging traditional industry boundaries and business practices.

Finally, the study by Shree *et al.*, 2021 highlights the multifaceted role of technological, organizational, and environmental contexts in shaping the adoption and success of digital platforms in B2B markets. Their analysis not only explains the technological affordances and organizational readiness that foster digital platform adoption but also underscores the environmental catalysts and inhibitors influencing such platforms' trajectory. Moreover, by charting out a future research agenda, the authors emphasize on the pressing need for empirical studies exploring the relationships between platform providers and users, the impact of digital platforms on business models, and the dynamics of value co-creation within B2B platform ecosystems.

### 2.2. Technology-oriented reviews

Emerging technologies like blockchain, data and mobile app marketplaces are reshaping the digital marketplace landscape. Tkachuk *et al.* (2021) and Azcoitia and Laoutaris (2022) offer systematic reviews on the adoption of blockchain in telecommunication services and the operation of data marketplaces, respectively. Similarly, another review from Abbas *et al.* (2021) examines the transformative potential of data marketplaces in catalyzing the data economy, while Petsas *et al.* (2013) provide critical insights into app popularity trends, download patterns, and the influence of economic models on app distribution and revenue generation.. These works utilize case study analysis and content analysis methods to explore the operational dynamics and challenges of technology adoption in marketplaces.

Notably, Tkachuk *et al.* (2021) provide a survey of Telecommunication Services Marketplaces (TSMs), emphasizing the role of blockchain as a trust-enabling mechanism that makes unnecessary the need for centralized intermediaries. This paradigm shift facilitates a more equitable value distribution among stakeholders, thus catalyzing innovation and streamlining service delivery processes. By analyzing the architecture and operational dynamics of blockchain-based marketplaces, the survey presents the potential of distributed ledger technology (DLT) to revolutionize the telecommunication sector's business models. Furthermore, the authors underscore the practical applicability and future prospects of blockchain in fostering a collaborative and transparent digital ecosystem for telecommunication services.

Azcoitia and Laoutaris (2022) survey presents the evolving landscape of Data Marketplaces (DMs), providing an overview of their business models, operational strategies, and the challenges they face. This work not only identifies a wide array of entities engaged in data trading but also categorizes them into distinct business models, underscoring the diversity and complexity inherent in the data economy. Moreover, the study highlights the significant role of technological innovations, such as blockchain and digital watermarking, in addressing critical issues related to data ownership, privacy, and value determination. Their analysis provides an understanding into how data marketplaces can speed up the growth of the knowledge economy, fostering an interconnected and efficient global data ecosystem.

Similarly, Abbas *et al.* (2021) provide a pivotal examination of the current research landscape through their systematic literature review. Their study emphasizes on computational pricing and architecture while revealing a shortage in empirical research on non-technical facets crucial for marketplace commercialization. By employing the Service-Technology-Organization-Finance (STOF) model as an analytical framework, they identify significant knowledge gaps, particularly in understanding market dynamics, user value, and governance structures that are essential for transitioning from platform design to adoption stages. Their call for a balanced inquiry into both technological and socio-economic dimensions of data marketplaces underscores the need for an academic and practical effort to realize their full potential in the emerging data economy.

Finally, the research of Petsas *et al.* (2013) challenges conventional assumptions about app popularity distributions, highlighting a significant deviation from the traditional Zipf-like behavior due to a phenomenon termed as the "clustering effect." This effect, attributed to users' propensity to focus on specific app categories over time, introduces a new dimension to understanding user behavior within app marketplace ecosystems. Furthermore, the authors offer a pioneering model for Appstore workloads, incorporating this clustering effect, which presents a closer approximation to real-world app download distributions than previous models. Their analysis extends to examining the consequences of app pricing strategies, shedding light on the varying financial outcomes for developers stemming from paid versus free app models. This study not only enriches the comprehension of the mobile app marketplace dynamics but also offers pragmatic insights for stakeholders in the app development and distribution chain.

### 2.3. Summary of related work

To better present the diverse methodologies, analytical perspectives, and key findings of seminal works in this domain, Table 1 presents a summarization. This consolidation aids in identifying the thematic continuities and differences within the literature, laying the groundwork for our research.

**Table 1.** Summary of related work on digital marketplaces.

Study reference	Research methods used	Data analysis	Major conclusions
Shirzad & Bell (2013)	Systematic literature review, Mixed- methods	Quantitative and Qualitative Analysis	Highlighted the importance of the TOES framework in understanding e-procurement marketplace flexibility. Emphasized a comprehensive approach to marketplace design and adaptation.
Cano *et al.* (2022)	Bibliometric analysis, Systematic review	Thematic Analysis, Bibliometric Techniques	Unveiled the focus on the relationship between e- marketplaces and open innovation. Highlighted the role of sustainability in shaping future marketplace models.
Täuscher & Laudien (2018)	Systematic literature review, Mixed- methods	Analytical Framework	Defined six distinct types of marketplace business models. Discussed their impact on innovation, competition, and regulation within digital ecosystems.
Shree *et al.*, 2021	Systematic review	TOE Framework, DOI Theory	Explored the role of technological, organizational, and environmental contexts in the adoption of digital platforms in B2B markets. Proposed future research directions.
Tkachuk *et al.* (2021)	Systematic review, Case study analysis	Qualitative Analysis	Emphasized blockchain's role in Telecommunication Services Marketplaces as a trust-enabling mechanism. Discussed the potential of DLT to revolutionize business models.
Azcoitia & Laoutaris (2022)	Systematic review	Qualitative Analysis	Presented the evolving landscape of Data Marketplaces, discussing challenges and the role of technological innovations.
Abbas *et al.* (2021)	Systematic review	STOF Model Analysis	Highlighted gaps in understanding market dynamics, user value, and governance structures in data marketplaces. Called for a balanced inquiry into both technological and socio-economic dimensions.
Petsas *et al.* (2013)	Systematic review	Statistical Analysis, Model Development	Challenged conventional assumptions about app popularity distributions. Introduced the "clustering effect" and its implications for appstore workload models.

### 2.4. Identified research gaps & contribution

As already mentioned in Section 1 and from what was mentioned in Section 2.1, Section 2.2 and Section 2.3, existing literature provides valuable insights into the business and technology aspects of digital marketplaces, however there is a notable gap in studies focusing on EU-funded marketplaces. This gap extends to an understanding of how these marketplaces leverage emerging technologies to address specific economic and societal challenges within the EU context.

This study aims to bridge the identified gap by conducting a systematic literature review of EU-funded digital marketplaces. By focusing on the integration of emerging technologies and examining their socio-economic impact, we contribute to the body of knowledge by offering an understanding of the role of EU funding in shaping innovative marketplace ecosystems.

## 3. Methods

As mentioned in the previous Sections, the current research work represents an SLR following the instructions for conducting a Systematic Literature Review as presented in the works of Kitchenham (2004) and Kitchenham, Charters, *et al.* (2007), adhering also to the PRISMA guidelines ^5^. Moreover, this research study was also inspired by the following works performing SLRs based on the above guidelines: a) the work of Spanos and Angelis (2016), who conducted an SLR in order to measure the impact of information security incidents on stock prices; b) the work of Polymeni *et al.* (2022), who performed an SLR on environmental Internet of Things (IoT) applications that implement prediction-based models; and finally c) the work of Alamdari *et al.* (2020), who conducted an SLR, focusing on e-commerce recommender systems.

Although the aforementioned methodology is widely used for conducting SLRs in the field of software engineering, the basic rules are more generic and can be easily applied in multiple research fields (evidenced by the three aforementioned research works), as it provides a clear and consistent approach for identifying and evaluating relevant literature, and for that reason, it was also followed in the present systematic review.

Based on these guidelines, the present SLR is comprised of three stages, as explained below:

(1) The *planning* stage, where the main research questions are defined acting as the basis on which the rest of the research work is conducted. The review protocol is also prepared in this stage.
(2) The *conducting* stage, where the selection of the primary studies takes place followed by the quality assessment of those studies.
(3) The *reporting* stage, where the results of the present SLR are circulated and communicated in a structured, visualized and comprehensive manner.

**Figure 1.**
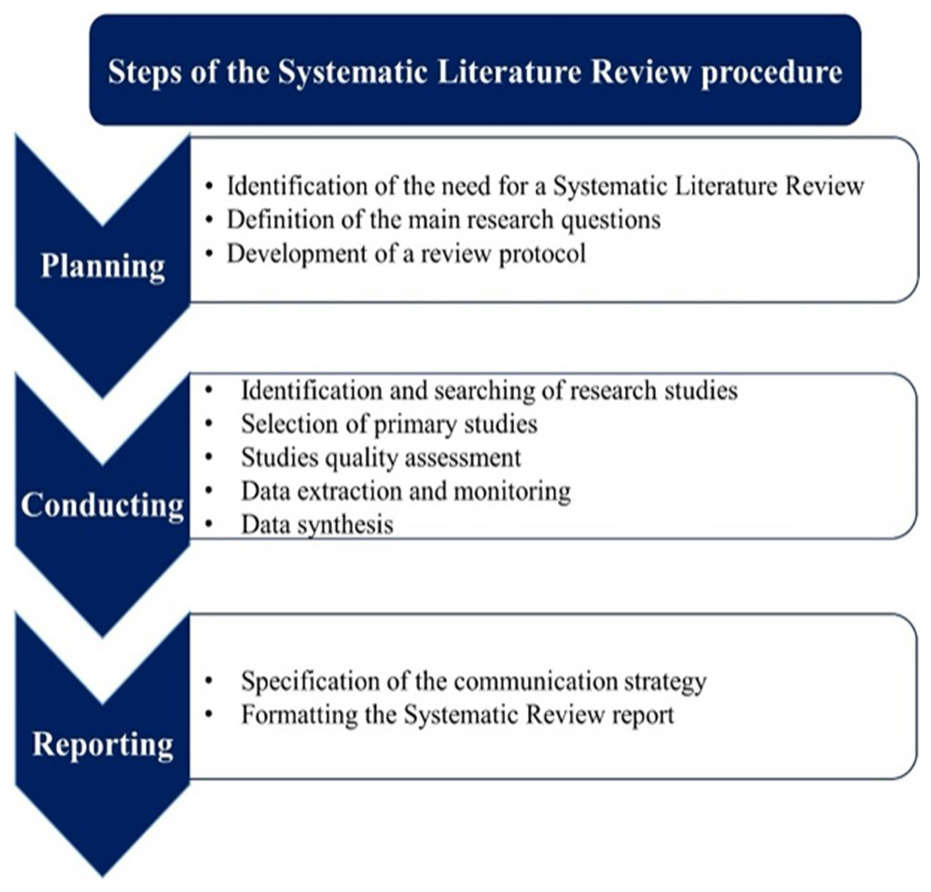
Steps of the Systematic Literature Review procedure.

The steps of the process are depicted in Figure 1, and will be thoroughly explained. The first stage of an SLR is the planning stage and consists of three steps. The first is the identification of the need for conducting the SLR. As already mentioned in the previous Sections, there are many research studies that aim to investigate the field of digital marketplaces in general, but to the best of our knowledge there is no review that specifically focuses on examining digital marketplaces developed through European funding and thus, presenting specific characteristics as mentioned in Section 1. Therefore, the need for the present SLR emerges from the requirement to examine and formulate a detailed analysis, in a complete and impartial manner ( Kitchenham, 2004), of different types of digital marketplaces delivered from European funding. The second step in this stage is the definition of the main research questions that need to be addressed through this research work and that affect almost every step of the whole procedure. According to the guidelines of Kitchenham (2004), the extraction of research questions is a very crucial step in order fulfill the research purpose of the SLR and to cover the research gaps, formulated in the Introduction, Moreover, the research questions can be refined and enhanced during the whole procedure studying the primary research works. Hence, the research questions that will be answered in this SLR are the following:

(1) What is the type of marketplace described in each study, based on their market offerings (product, software, services, etc.)?
(2) What is the sector/industry that the marketplace was designed to operate in?
(3) Is the marketplace blockchain-based or not?
(4) What are the main technologies utilized for the marketplace development in the literature?

The last step involved in the planning stage is the development of the review protocol that actually defines the whole procedure that is followed in this SLR. It essentially describes all the actions necessary to proceed in the conducting stage. These steps are described next and namely are the following: a) finding the query that will be used for the selection of the primary studies in the scientific databases, b) the definition of the inclusion/exclusion criteria, c) the selection of the quality assessment criteria, and lastly d) the definition of the main data features that will be obtained from each study and are useful for the proposed SLR. Table 2 presents the query that was used in this study. The terms added to the query were searched in the title, abstract, keywords of each study, and also in the funding-related details, since the goal of the present SLR is to only include studies that present a digital marketplace that was developed in the course of an EU-funded project. The selection of the right and most accurate search string is an iterative procedure, since the aim is to extract as many related primary studies as possible in an accurate and rigorous way ( Kitchenham *et al.*, 2007). Finally, in this step, the scientific databases were determined following a similar consideration with other SLRs ( Polymeni *et al.*, 2022; Spanos & Angelis, 2016) and these are: Scopus, IEEE, ACM, and Web of Science.

**Table 2.** Search query structure: (marketplace-related results) AND (funding-related results).

*(ABS ("marketplace") OR TITLE ("marketplace") OR KEY ("marketplace")) AND (FUND-ALL ("EUROPEAN") OR FUND-ALL (EU) OR FUND-ALL (EC))*

The backward and forward snowball techniques were also used as supplementary methods in order to expand the search and to find relevant research studies that could not be found through the first method ( Ketsetsis *et al.*, 2020). The guidelines followed for the snowball technique are the ones proposed by Wohlin (2014), who describes how to conduct literature reviews using the snowballing technique. For this SLR the automated search in scientific databases as proposed by Kitchenham (2004) and ( Kitchenham *et al.*, 2007) is the primary methodology used, but in order to ensure that this review paper covers the clear majority of studies in the research field, a combination with the snowball technique was performed. The backward snowball technique refers to using the reference list of each study in order to identify new papers in an iterative manner. After each iteration, new related papers might be found, and when no more new related studies can be found the procedure ends. The forward snowball technique refers to searching for papers citing the paper being examined. The citations are studied by using Google Scholar. The approach to go through each paper is iterative and similar as in the backward snowball technique. At this point, it is worth mentioning that following the aforementioned methodology for the automated search of relevant research studies, also used in the vast majority of SLRs, the grey literature was not considered, although that for the digital marketplaces of EU-funded projects, there are many technical reports (grey literature) explaining their full functionality. This exclusion in the current SLR was performed, apart from being aligned with the majority of SLRs, in order to investigate the research footprint of these marketplaces, which can be better depicted by studies published in research journals and conferences.

In every SLR, the inclusion and exclusion criteria must be transparent and clearly stated, as they make the primary study selection procedure easier. The following criteria were used in the present SLR for the selection of the primary studies to be examined:

- **Inclusion Criteria:**
  - Studies published from 2013 until 2023
  - Studies in which a fully-developed or just a prototype of a digital marketplace is presented and is also funded from European grants as part of a research and/or innovation Programmes
- **Exclusion Criteria:**
  - Short papers
  - Review studies and surveys
  - Papers not written in English language
  - Studies that present an implemented marketplace but are part of a national project
  - Studies that do not present an implemented marketplace, but rather describe the concept or the idea of a digital marketplace

The quality assessment criteria are an essential part of the SLR procedure, as they are defined to set the limits and ultimately ensure that all studies included in the SLR, after the full content scan, accomplish an adequate level of quality. When it comes to quality assessment criteria in software systems such as digital marketplaces, the classification of such criteria becomes a little bit unclear compared to model-based studies. For this reason, a similar consideration with the SLR of Polymeni *et al.* (2022) was followed and according to this approach, the following criteria are considered for quality assessment in the present SLR work:

- **System description**: each study gives a clear overview of the marketplace presented and specifically focuses on some or all of its functionalities.
- **Business and technical requirements**: every paper included in the SLR gives at least a generic overview of what each marketplace is supposed to do (business aspect) and how it is supposed to do it (technical aspect).
- **Business impact**: each study provides at least some general information regarding the business goals and the impact they want to bring into the industry that the marketplace will operate in.

After having defined all of the aforementioned actions and steps, the last necessary step for the development of a solid review protocol is the selection of the data features that will be obtained from the papers found in the search and will prove useful for the present SLR work. These data features are presented and explained in Table 3.

**Table 3.** Data features to be extracted from each primary study.

Data Features	Description
Authors	The authors that participate in the study.
Year	The publication year of the paper.
Publication source type	The source type of the study (i.e. conference, journal).
Publication source	Publication source of the paper.
Sector/industry	The classification of each marketplace presented in each study to the industry they were designed to operate in.
Marketplace type	The categorization of each marketplace from every paper to a certain type based on its market offerings (e.g., product, software, service, etc.).
Marketplace business targets and objectives	A brief description of the primary objective and area of emphasis of every digital marketplace.
Payment method (if described)	It refers to whether each paper also describes how payments are handled in the marketplace.
Blockchain-based	Describes if the marketplace is blockchain-based or not.
Blockchain details	For every blockchain-based marketplace, the related technologies are documented.
Architecture visualization	Describes if each study also provides a figure of the architecture behind every marketplace.
Existence of UI screenshots	It portrays if there are any UI screenshots included in the papers or not.
Technologies used	The main technologies (if provided) used to develop and implement the marketplace from each study. Every technology described will be part of a broader category (e.g., computer languages, frameworks, etc.).
Link for open-source repository	It presents if the papers examined also provide the link for an open-source repository (e.g., GitHub).
Link for marketplace website	It refers to whether each study also includes the link to the marketplace website or not.
Major research outcomes	It describes in a brief manner the major research topics and major conclusions drawn from each study.

**Figure 2.**
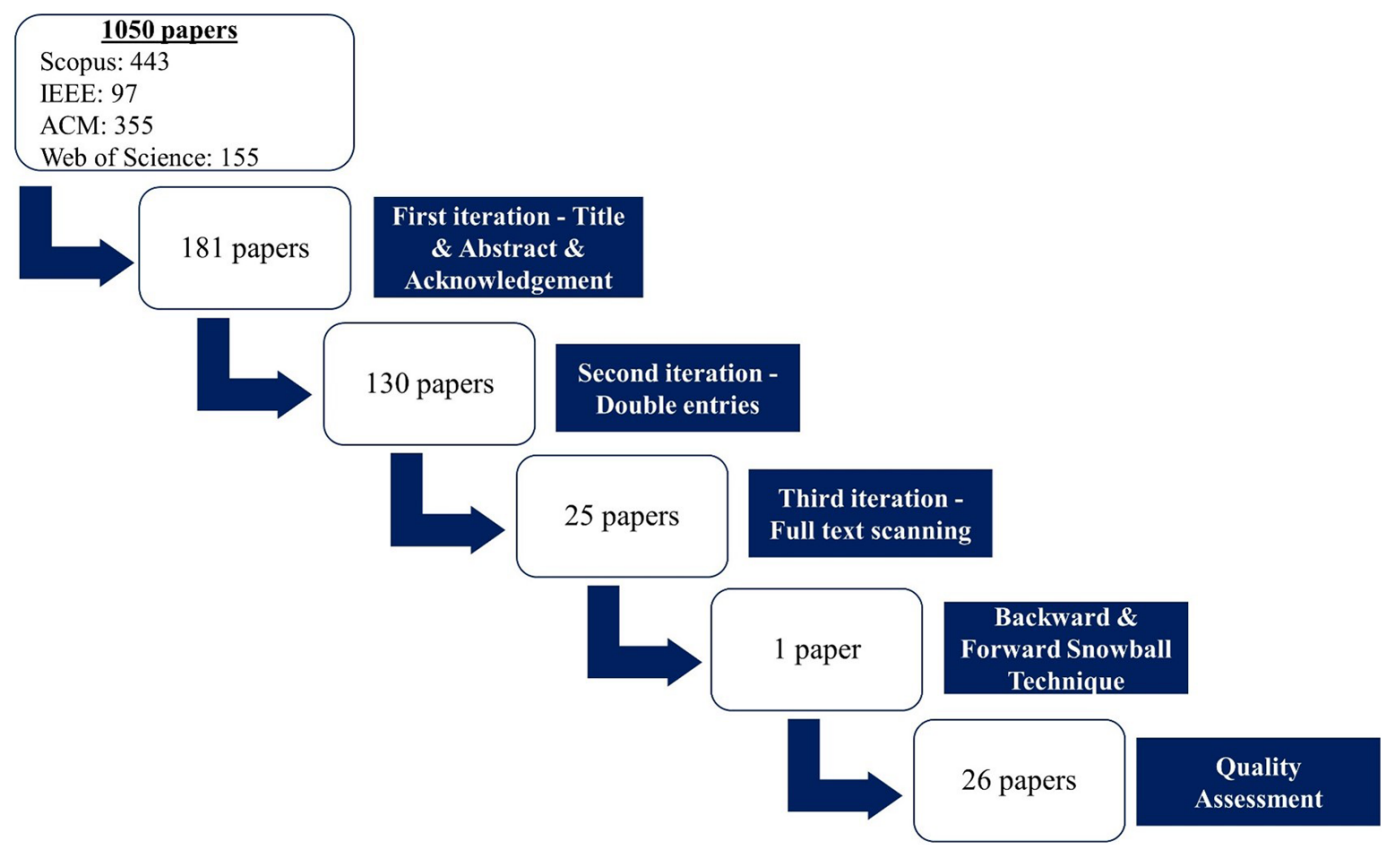
Flowchart with the steps for the systematic literature review procedure.

The execution of all the predefined steps described in the SLR methodology is the next procedure that follows after the development of the review protocol. As Figure 2 depicts, during the first search process 1050 papers were found (443 from Scopus, 97 from IEEE, 355 from ACM and 155 from Web of Science). The execution of this step is then followed by scanning the title, abstract, acknowledgements, and funding details since the goal of the present SLR work is to determine if the marketplace presented in each paper is delivered through European funding or not. In this step, 181 papers are included, while the rest are excluded. After this step, the exclusion of the double entries follows leaving the total number of included papers at 130. The final step before proceeding to the quality assessment process is the full-text reading of every paper. The number of papers remaining after this step is 25.

As noted earlier, and in order to ensure a comprehensive review of the literature, the backward and forward snowballing techniques were employed to identify relevant studies that were not found in the initial search process. After this additional step, one more paper meeting the inclusion criteria was identified, bringing the total number of papers in this systematic literature review to 26. All of the studies included in this review fully met the quality assessment criteria previously defined and therefore, the final number of examined research works in the current SLR is 26.

## 4. Results

This Section presents the results of this systematic literature review that answers the four research questions set in the previous Section. As already noted, the final number of the included papers is 26, and the results gathered from each study are categorized and presented into the three following subsections: *General Results*, *Business-related Results*, *Technical-related Results and Research-related Results*.

### 4.1. General Results

**Figure 3.**
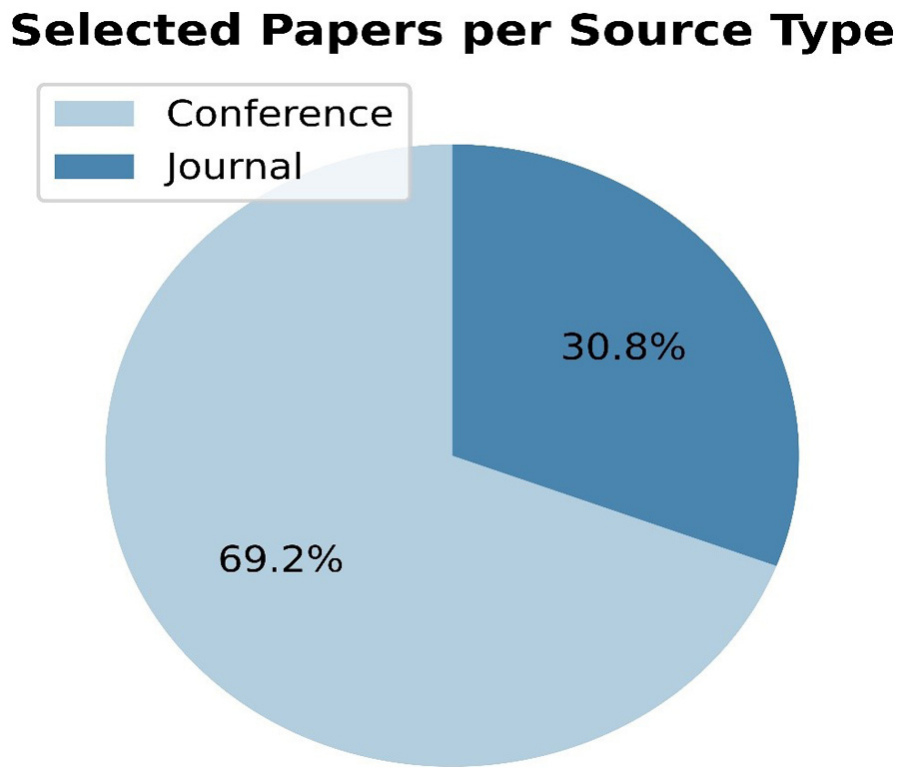
Pie chart depicting the percentage per source type.

In this Section, the general results extracted from the selected primary studies are presented. Specifically, we focus on two categories of data: (a) publication characteristics, which include information on authors, publication sources, and source types; and (b) the distribution of the selected primary studies per year. Table 4 summarizes the publication characteristics of the 26 primary studies. The majority of the papers included in this study (18 out of 26) and as Figure 3 depicts, were published in conference proceedings, while the remaining 8 papers were published in journals. These results indicate that a significant proportion of the studies were more preliminary and exploratory in nature, as conference papers often feature early results or works in progress. Conversely, a third of the papers analyzed were more comprehensive and rigorously researched, which is a hallmark of journal publications. Hence, it is worth mentioning that due to this proportion of papers between conference and journal papers, the present statistical analysis of the results requires careful treatment, and the generalization of these results is not always feasible. However, toward the extraction of meaningful research findings, apart from the statistical analysis, a qualitative analysis is performed in the current manuscript.

**Table 4.** Publication characteristics of the selected primary studies.

No.	Authors	Publication Source	Source Type	Year
a1	Ali *et al.* (2013)	CCIS	Conference	2013
a2	Stavropoulos *et al.* (2020)	AISC	Conference	2020
a3	Westera *et al.* (2019)	LNCS	Conference	2019
a4	Roman *et al.* (2017)	CEUR Workshop Proceedings	Conference	2017
a5	Barmparitsas *et al.* (2022)	SEEDA-CECNSM	Conference	2022
a6	Pincheira *et al.* (2020)	SDS	Conference	2020
a7	Faliagka *et al.* (2022)	VLSI, ISVLSI	Conference	2022
a8	Alvsvåg *et al.* (2022)	CCIS	Conference	2022
a9	Genés-Durán *et al.* (2022)	SCC	Conference	2022
a10	Bassiliades *et al.* (2018)	Data and Knowledge Engineering	Journal	2018
a11	Shi *et al.* (2022)	Journal of Cloud Computing	Journal	2022
a12	Menychtas *et al.* (2014)	Future Generation Computer Systems	Journal	2014
a13	Niya *et al.* (2021)	IFIP/IEEE IM	Conference	2021
a14	Maciel *et al.* (2019)	NetSoft	Conference	2019
a15	Horsch *et al.* (2020)	KI - Kunstliche Intelligenz	Journal	2020
a16	Palaiokrassas *et al.* (2021)	Computers	Journal	2021
a17	Fernández-Fernández *et al.* (2021)	EuCNC/6G	Conference	2021
a18	Fotiou *et al.* (2021)	Blockchain: Research and Applications	Journal	2021
a19	Bernardinetti *et al.* (2021)	ARES	Conference	2021
a20	Markakis *et al.* (2019)	IEEE Communications Magazine	Journal	2019
a21	Oikonomou *et al.* (2021)	CSR	Conference	2021
a22	Mladenov *et al.* (2022)	Energies	Journal	2022
a23	Rehm *et al.* (2021)	EACL	Conference	2021
a24	Bavota *et al.* (2014)	CSMR-WCRE - Proceedings	Conference	2014
a25	Gönül *et al.* (2023)	I-ESA	Conference	2023
a26	Mavrogiorgou *et al.* (2022)	ESSE	Conference	2022

It is also notable that only two of the primary studies reviewed (7.69% of the total) were published in the same conference proceedings (Communications in Computer and Information Science - CCIS), while the others were published in different sources, reflecting that way the multi-disciplinary nature of this research. Finally, it is worth noting that only one author appears as an author in more than one of the included research studies.

**Figure 4.**
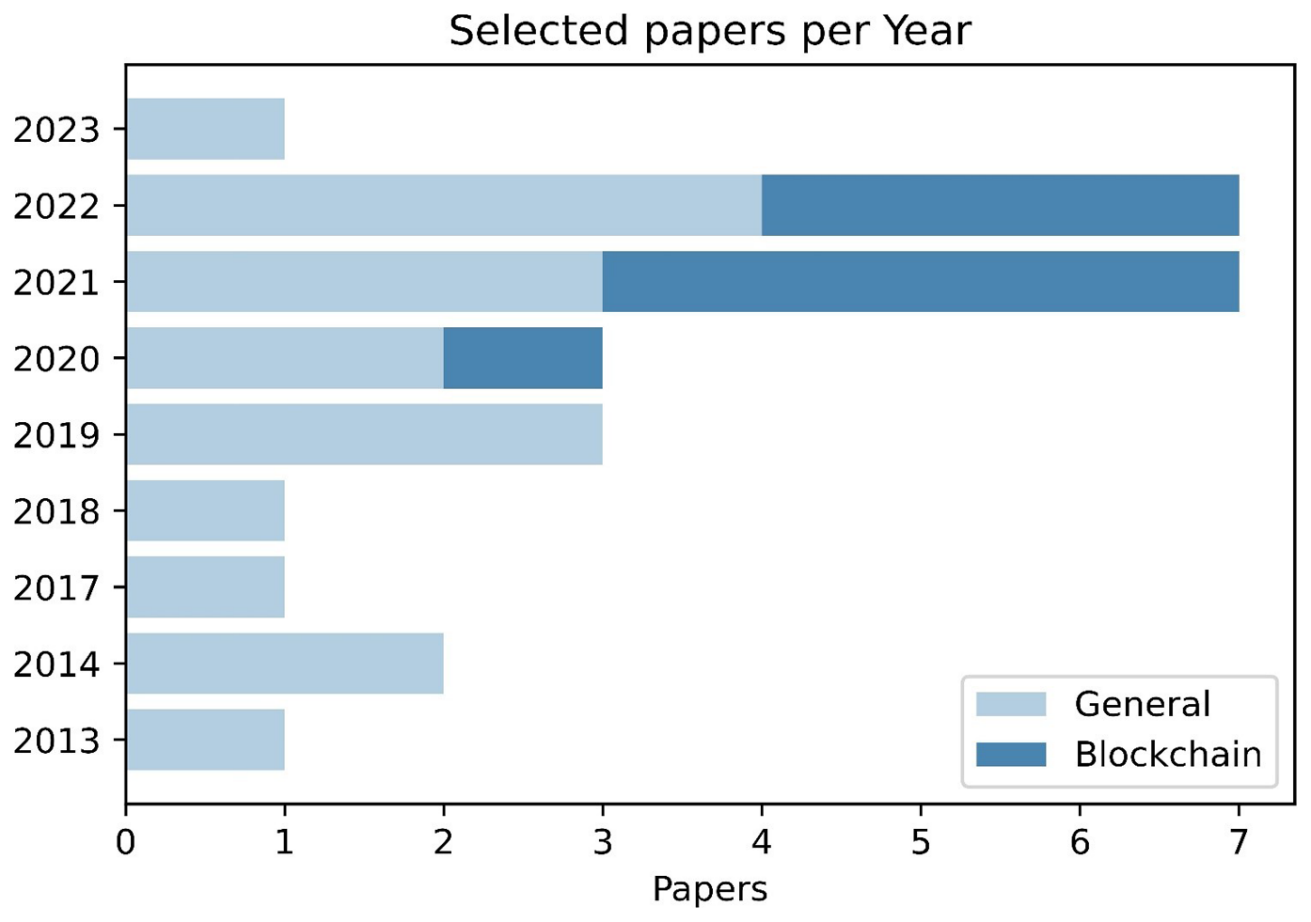
Column chart depicting the papers per year and the blockchain papers per year.

Table 4 yielded another significant observation concerning the quantity of primary studies published each year. As shown in Figure 4, only 4 out of 26 studies (approximately 15.38%) were published from 2013 until 2017 while the vast majority (22 out of 26) of the papers (approximately 84.62%) were published from 2018 until 2023. The observed difference can be attributed to the growing number of digital marketplaces funded by European research and innovation programs, which leverage the collaboration and interconnectivity capabilities of these platforms. By sharing, offering, and managing their market offerings through these platforms, partners, and stakeholders can effectively exploit the benefits of digital marketplaces. Figure 4 also aims to gauge the blockchain’s impact on the deployment of marketplaces. Blockchain was part of the reviewed marketplaces after 2020 and continues to gather interest. The publication date for including blockchain is strongly associated to the advent of smart contracts, open-source solutions, and their corresponding inclusion in the proposal of European projects. Smart contracts ( Kolvart *et al.*, 2016) were introduced in 2016 and are the catalyst for blockchain to enter the next generation. The importance and resulting inclusion of smart contracts into European projects logically lag in time due to the procedures for a proposal to be accepted as a project and deliver results. Blockchain is included in 4 and 3 papers for 2021 and 2022, respectively, numbers comparable to the selected papers per year. The research community will continue to experiment with the use of blockchain as it can be a versatile tool for security, auditability, and transparency for marketplaces.

### 4.2. Business-related Results

This Section presents the results of the business-related analysis from the SLR. This analysis aims to provide a comprehensive understanding of the various types of digital marketplaces presented in each primary study and their applicability to different industries. In addition, the business-related analysis also includes some specific details for the business targets and objectives for each marketplace. This information adds another layer of understanding and sheds light on how these marketplaces operate. Overall, this Section examines in detail the current state of digital marketplaces in terms of their business aspects and highlights trends and potential areas for future research. In this study, the classification of every marketplace to a certain type was based on their market offerings or to be more precise on the type of goods or services they offer. From the literature analysis, the identified types of marketplaces were classified into the following categories:

(1) **Software Marketplace**: Marketplaces that offer software applications, such as mobile, desktop, and web apps.
(2) **Data Marketplace**: Marketplaces that provide various types of data sets, such as weather data, through a digital platform.
(3) **Product Marketplace**: A type of marketplace that specializes in facilitating the exchange of physical goods and/or serving as an intermediary for procuring such products.
(4) **Services Marketplace**: Marketplaces that enable individuals, professionals, or companies to offer their services online.
(5) **Multi-typed Marketplace**: A marketplace type that combines two or more of the attributes of the above types of marketplaces.

Regarding the classification of every marketplace to the industry they were designed to operate in, it was also based on the type of goods or services offered that are related to various domains, such as cloud computing, healthcare, automotive, manufacturing, among others. Hence, this was the primary criterion used to categorize the industry of each marketplace in the primary studies. However, this categorization was not always strict, as many marketplaces could operate and be applicable in more than one industry. In cases where a marketplace did not have a specific industry focus, the term ”Generic” was used to classify it. In addition to the previous data features, this Section includes a brief overview of the primary objective and area of emphasis of each marketplace, thus providing further insight into the business goals that each marketplace aims to accomplish. Table 5 highlights the overall results of the business-related analysis regarding the aforementioned data features that were extracted and gathered from the selected primary studies.

**Table 5.** Categorization of each marketplace from every primary study per type and per industry.

No.	Industry	Marketplace type	Marketplace business targets and objectives
a1	Generic	Software	A marketplace offering cloud services to business customers that come attached with verifiable certificates of security properties.
a2	Healthcare, IoT	Software	This one-stop-shop brings together business entities/developers with end-users/health professionals by offering various IoT apps in the field of Active \& Healthy Ageing (AHA).
a3	Gaming	Multi-typed	This multi-typed marketplace is designed to become a knowledge hub and a digital repository in the gaming community where relevant software products and tools can also be exchanged.
a4	Generic	Data	The platform enables data providers to publish, distribute and monetize their data and simultaneously, facilitates data consumers in discovering and accessing data available on the marketplace.
a5	Circular Economy, HVAC-R	Product	Aims to change the way the industry handles used F-gases by supporting the circular economy of refrigerants in the HVAC-R sector.
a6	Cloud Computing, IoT	Services	A distributed and decentralized marketplace designed to deliver computational, storage and network services.
a7	Generic	Multi-typed	This multi-typed marketplace offers a variety of solutions (across various domains), such as tools, matchmaking services, funding opportunities, and educational material.
a8	Urban Planning	Data	A data marketplace designed to not only support the buying and selling of data but also foster knowledge and experience sharing among the users, in smart and sustainable cities.
a9	Automotive, Manufacturing, Healthcare	Data	The primary objective is to establish equitable competition and trust among various stakeholders, promoting the development of a digital-data trading ecosystem in Europe.
a10	Cloud Computing	Services	A Platform as a Service (PaaS) marketplace addressing the challenges of cloud platform compatibility and the transferability of cloud applications.
a11	Cloud Computing	Multi-typed	A decentralized auction-based platform for the exchange of software and data in a trustworthy ecosystem.
a12	Cloud Computing	Multi-typed	This marketplace aims to act as a market in the context of Anything as a Service (XaaS), in order to foster the creation of viable business ecosystems.
a13	IoT	Data	A blockchain-based IoT data marketplace to facilitate the access to a wide range of data providers in a manner that is both simple and compliant with privacy regulations.
a14	Cloud Computing	Services	A services marketplace that aims to foster slice resource discovery and information exchange.
a15	Manufacturing	Multi-typed	A marketplace for the exchange of data, software and services related to material modelling.
a16	Urban Planning, IoT	Data	A blockchain-based data marketplace for the exchange of IoT sensor data and media in an easy, secure and anonymous way.
a17	Telecommunications	Services	A services marketplace based on blockchain and designed to enable secure trade of various resources amidst the fast-evolving 5G technology landscape.
a18	IoT	Data	A data marketplace based on blockchain that enables data consumers to acquire noisy data that can be utilized for extracting meaningful statistics.
a19	Cybersecurity	Services	This marketplace aims at fostering the exchange of knowledge regarding cyber range services.
a20	Cybersecurity	Services	This marketplace platform offers a variety of cutting-edge cybersecurity services, introducing novel business scenarios and significantly broadening potential markets.
a21	Cybersecurity	Services	A marketplace acting as an intermediary between customers and providers offering cyber range services.
a22	Energy	Services	A marketplace acting as a flexibility market between Transmission System Operators (TSOs), Distribution System Operators (DSOs), and Prosumers in a secure, transparent, cost-effective manner.
a23	Language Technology	Multi-typed	This multi-typed marketplace aims at enabling the upload, share, and distribution of software, products, and resources in the European Language Technology Landscape.
a24	Generic	Multi-typed	A multi-typed marketplace that aims to act as a public directory and search engine of free and open source software fostering the exchange of knowledge in the community.
a25	Generic	Product	A B2B marketplace addressing challenges in semantic annotation and introducing a generic product classification taxonomy model using eClass and Furniture Sector Taxonomy as examples.
a26	Generic	Data	A cross-domain marketplace integrating diverse solutions and assets, with a focus on storage, advanced search, retrieval, and combining data assets into comprehensive offerings.

**Figure 5.**
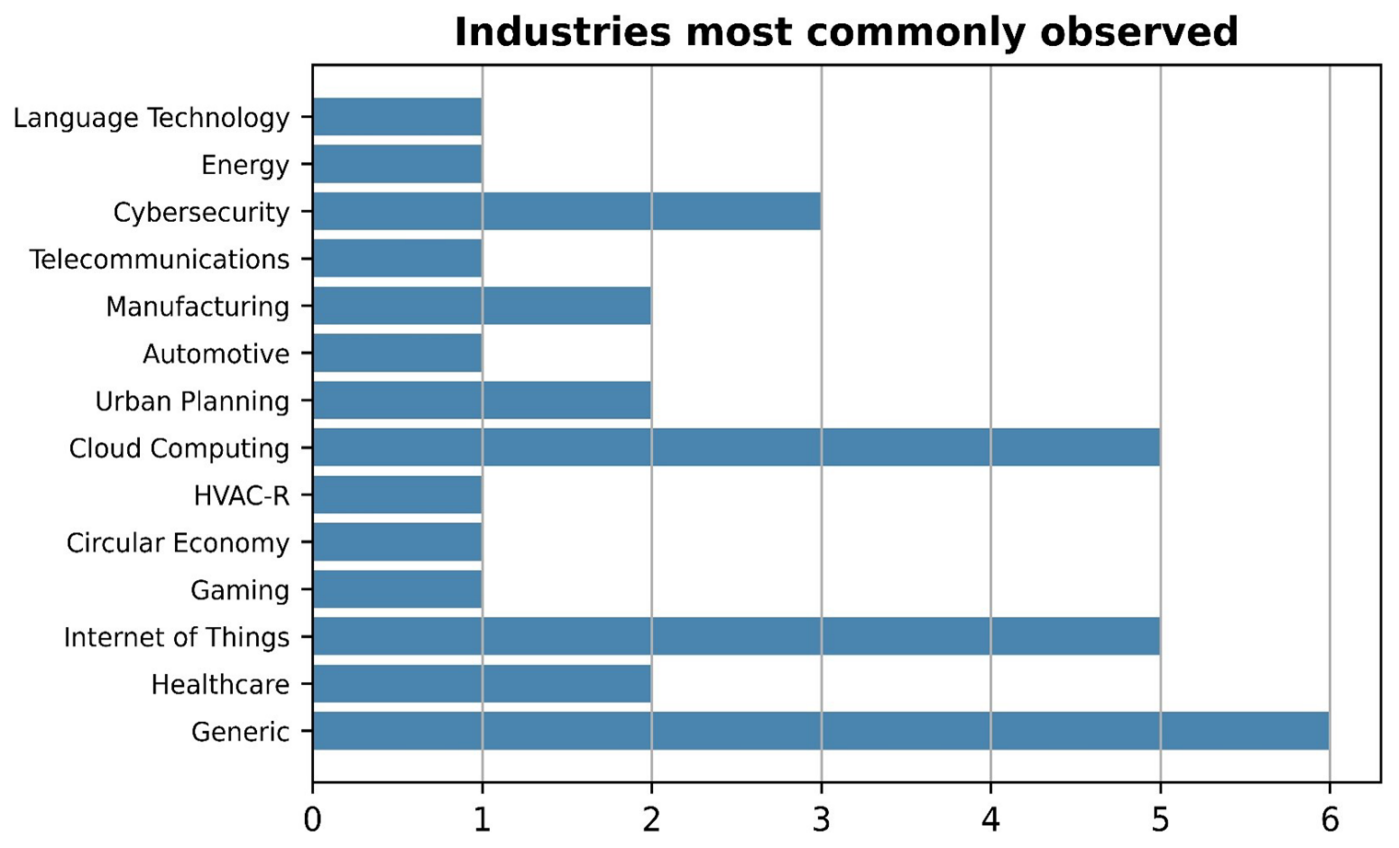
Column chart depicting the most frequently encountered industries.

Based on the analysis of Table 5 and as depicted in Figure 5, it was observed that most marketplaces (6 out of 26) had a more generic nature making them suitable for a wide range of domains, which could explain their popularity. The generic nature of a marketplace enables the exploration of synergies, the creation of new value propositions, and the realization of cross-domain innovations by connecting complementary products and services from multiple industries. Furthermore, our analysis showcased that the Cloud Computing and Internet of Things industries were found to be the second most commonly encountered industries, accounting together for one-third of the total number. This could be attributed to their growing importance and increasing adoption across domains, since the demand for cloud computing services and IoT-enabled devices has been on the rise, and this trend is expected to continue in the foreseeable future ( Taha *et al.*, 2020). As a result, many entrepreneurs, businesses, and researchers are exploring opportunities to develop marketplaces that cater to these industries. Finally, Cybersecurity emerged as the next most frequently occurring industry sector (3 respective marketplaces that specifically focus on ensuring the uninterrupted operation of critical systems and services). All the remaining industries showcased in Figure 5 were identified only once or twice.

**Figure 6.**
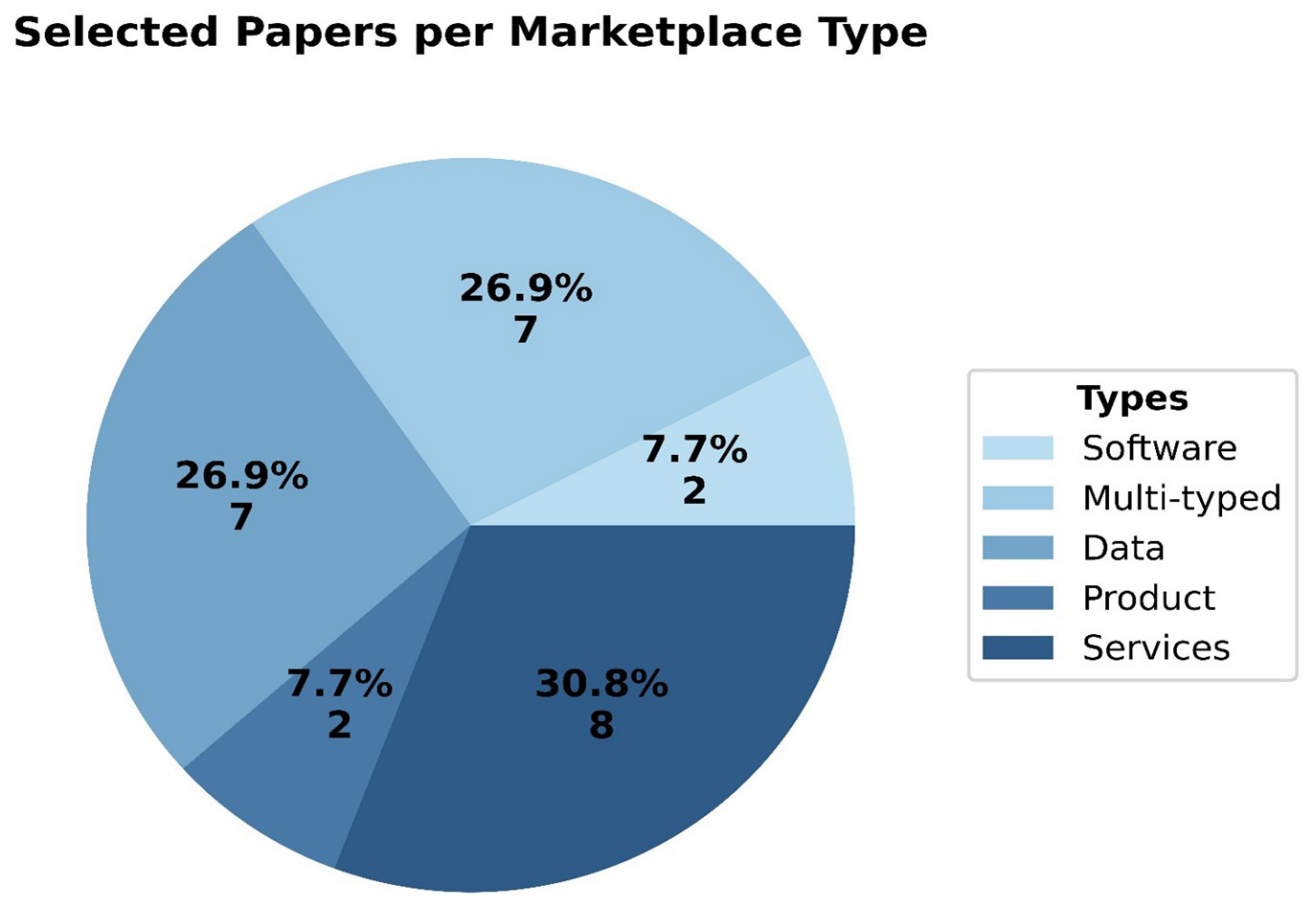
Pie chart depicting percentage per marketplace type.

In Figure 6, the classification of each platform into a marketplace type is presented. The results indicate that approximately one-third (30.8%) of the marketplaces are categorized as services marketplaces, either serving as intermediaries between service providers and consumers across various domains, or offering their own specific services. Another significant finding is that 26.9% of the marketplaces are multi-typed, providing a variety of software products, data sets, tools, services, and related items. This suggests an increasing trend toward hybrid marketplace models in multiple economic sectors. Additionally, the same percentage (26.9%) of the digital marketplaces are categorized as data marketplaces, with the majority of the encountered data marketplaces emerging after 2018, indicating a notable trend in the growing recognition and significance of data handling and distribution. Data marketplaces offer opportunities for businesses to unlock the untapped potential of data, foster collaborations, and leverage data-driven insights to drive growth and competitive advantage. Notably, only two marketplaces are classified as software marketplaces, and only two as well exclusively deal with product trading among stakeholders and across domains. All the above, highlight the current trends regarding marketplace type (services and data) in electronic marketplaces, resulting also in the fact that traditional product marketplaces, at least for the EU-funded projects, are of minor scientific importance and somehow old-fashioned, therefore eliminating geographical barriers and enabling convenient access to a wide range of products and services.

***4.2.1. Payment and pricing.*** The addition of "Payment and Pricing" as a distinct subsection within the broader context of business-related results stems from its critical role in shaping the operational and economic landscape of these platforms. Despite the exploration of types, industries, and business objectives of digital marketplaces presented earlier, the specific mechanisms of payment and pricing have a direct impact on marketplace sustainability, user adoption, and overall financial health. In spite of its critical importance, it was observed that only a limited number of papers within the systematic literature review explicitly investigate payment and pricing mechanisms. This selective coverage highlights the need for a focused examination to understand the subtle differences and impacts of these financial strategies on the digital marketplace ecosystem.

The payment and pricing methods used in digital marketplaces are a crucial aspect of their business model. However, the proposed SLR found that only one-third approximately of the 26 primary studies analyzed provided a detailed analysis of the payment methods used, limiting the ability to conduct a detailed and comparative analysis of this parameter. While this is a limitation, it is important to acknowledge that the primary studies did not always focus on business-related aspects of the marketplaces, and therefore may not have provided a dedicated analysis of the payment method. Overall, payment and pricing methods are essential components of the business model of digital marketplaces. The present analysis highlights the variety of payment methods used in non-blockchain marketplaces and the potential of blockchain technology to introduce digital tokens and decentralize the agreement mechanism.

There are papers in the literature detailing a specific case like subscription ( Roman *et al.*, 2017), while others cover a broader spectrum of payment methods with four different methods ( Menychtas *et al.*, 2014). A degree of flexibility for adopting various methods can help marketplaces’ adoption, as elaborated in Alvsvåg’s future work ( Alvsvåg *et al.*, 2022). Additionally, the business model for the marketplace has room to contemplate the payment means. Indeed, Europe has introduced the Single Euro Payments Area (SEPA) network, and marketplaces can benefit from cashless euro payments ( Barmparitsas *et al.*, 2022). Finally, the business model can dictate technical requirements to marketplaces for adopting various agreement mechanisms. For example, Barmparitsas *et al.* (2022) describe that the trade and payment mechanisms are based on a Bidding model quite different from a traditional Auction Model, as only one Bid can be submitted per Listing and grade.

For blockchain-based marketplaces, the use of blockchain technology provides promising opportunities to decentralize processes and introduce trust into a network without a central authority. Due to the monetary nature of marketplace transactions, digital tokens can serve as a means for exchanges between buyers and sellers. In the research works examined, there are European marketplaces allowing users to select between fiat methods ( Genés-Durán *et al.*, 2022; Mladenov *et al.*, 2022), and digital token payment ( Genés-Durán *et al.*, 2022; Mladenov *et al.*, 2022; Palaiokrassas *et al.*, 2021). The papers introduce a native to the marketplace token by applying market-tested standards such as ERC-1155 ( Genés-Durán *et al.*, 2022) or ERC-223 ( Palaiokrassas *et al.*, 2021). Other works ( Fernández-Fernández *et al.*, 2021) abstract the notion of money to a numerical representation without specifying the currency to any digital or fiat one. Digital tokens may not be essential for the suggested applications as traditional payment methods remain applicable, and the application’s scope can differ from the payment method by suggesting other mechanisms. Apart from the payment, blockchain can decentralize the agreement mechanism. There are auction and bidding mechanisms accompanying the marketplaces, and there are ideas for automating the procedure with the use of smart contracts ( Shi *et al.*, 2022). Blockchain has the potential to decentralize procedures, but business models may require a revision. The business model should account for transactional settlement between individuals rather than devising a business case relying on central authorities. Business cases where the marketplace’s operator distributes the funds to the owners ( Fotiou *et al.*, 2021), confine the decentralization of the technology.

### 4.3. Technical-related Results

In this subsection of the SLR, a comprehensive overview of the technical aspects of the digital marketplaces from the selected primary studies is presented. The analysis of the technical features is a three-layered analysis including the following technical characteristics: a) the deployment-related features that examine the evidence and realization characteristics of the digital marketplaces; b) the classification of software technologies to broader categories based on the similarities or shared characteristics; and finally, c) the blockchain-related analysis that presents the technical features that are closely related to the blockchain technologies used in several marketplaces. The presentation of the technical features of every digital marketplace studied, allows for a deeper understanding of the digital marketplace ecosystem and can offer valuable insights that can help future research and provide guidance to practitioners and policy makers.

***4.3.1. Analysis of Deployment-related features.*** As mentioned above, the first layer of the technical analysis is the presentation of the deployment related features that examine the maturity development level of each marketplace at the time having been presented and provide insights concerning the availability and accessibility of the marketplace. The information extracted from each primary study are presented in Table 6. Although the deployment features are presented as Boolean values (Yes/No), they allow for a clear, concise and simplified analysis, since they offer an easy comparison among the different marketplaces. This approach can also highlight any potential issues or areas for improvement in the deployment of these marketplaces.

**Table 6.** Deployment related features for each primary study.

No.	Architecture visualization	UI screenshots	Link for open-source repository	Link for marketplace website
a1	No	Yes	No	No
a2	Yes	Yes	No	Yes
a3	No	Yes	No	Yes
a4	Yes	No	No	No
a5	No	Yes	No	Yes
a6	Yes	No	No	No
a7	Yes	No	No	Yes
a8	No	Yes	No	No
a9	No	No	No	No
a10	Yes	No	No	No
a11	Yes	Yes	Yes	No
a12	Yes	Yes	No	No
a13	Yes	No	No	No
a14	Yes	No	No	No
a15	No	Yes	No	No
a16	Yes	Yes	Yes	No
a17	Yes	No	No	No
a18	No	No	Yes	No
a19	No	Yes	No	No
a20	No	Yes	No	No
a21	Yes	Yes	No	No
a22	No	Yes	No	No
a23	Yes	Yes	Yes	Yes
a24	Yes	No	No	Yes
a25	No	Yes	Yes	No
a26	Yes	Yes	No	Yes

It is interesting to observe in Table 6 that out of the 26 primary studies analyzed, 57.69% (15 studies) have provided a visualization of the marketplace architecture. The distribution of architectural designs was found to be diverse across the 15 included studies. Architectural designs ( Hofmeister *et al.*, 2000) refer to the fundamental structure of a digital marketplace, which might include its components, modules, subsystems, infrastructure, and interfaces. From those 15 studies, multitiered architectural designs were utilized in the 33.33% (5 studies), while a more traditional component-based architecture was used in the 20% (3 studies). Service-oriented architecture ( Valipour *et al.*, 2009) was observed in the 6.67% (1 study), while the microservices architecture ( Ghofrani & Lübke, 2018) was utilized in the 13.33% (2 studies). Finally, 4 studies (26.67%) have presented a visualization of their architecture and belong to the blockchain-based marketplaces in which a decentralized architecture was observed and can be considered a distinct category of architecture, as it differs significantly from traditional centralized or client-server architectures, since there is no central authority or intermediary controlling the network, but instead, the system is distributed among multiple nodes or devices. Based on the above results, the information regarding architecture can be critical in understanding the overall structure and design of each marketplace, and in identifying the different components and the interactions that occur. Additionally, it can help identify the level of complexity involved in the implementation of the marketplace and the type of infrastructure required to support it. Overall, the visualization of architecture constitutes a valuable tool in software engineering life cycle offering insight into the technical aspects of the marketplace, thus making it also an essential component of the present SLR study.

Regarding the rest of the results presented in Table 6, out of the 26 primary studies analyzed, 61.53% (16 studies) provided user interface (UI) screenshots. From those 16 studies, in 4 studies (25%) the screenshots were limited to the homepage, in 5 studies the screenshots focused on specific functionalities or services provided by the platform (31.25%), and in the majority of the studies both the homepage and some of the marketplace functionalities are presented in the screenshots (43.75%, 7 studies). The inclusion of UI screenshots is important as it provides insights into the overall user experience and helps to identify potential usability issues and areas for improvement.

On the other hand, only 7 studies (26.92%) provided a link to the marketplace website, while only 5 studies (19.23%) provided a link to an open-source repository. The provision of website links and open-source repositories can greatly facilitate the evaluation and replication of the marketplace, with the latter also being useful for evaluating the implementation details and potentially reusing the source code. Furthermore, providing access to the marketplace website and open-source repository, in addition to the UI screenshots, can significantly enhance the transparency and reproducibility of the research results. Finally, these two features are also crucial for exploitation reasons of the EU-funded projects, since they prove the existence and operation of a marketplace after the finalization of the project and thus, their usefulness for the community.

**Figure 7.**
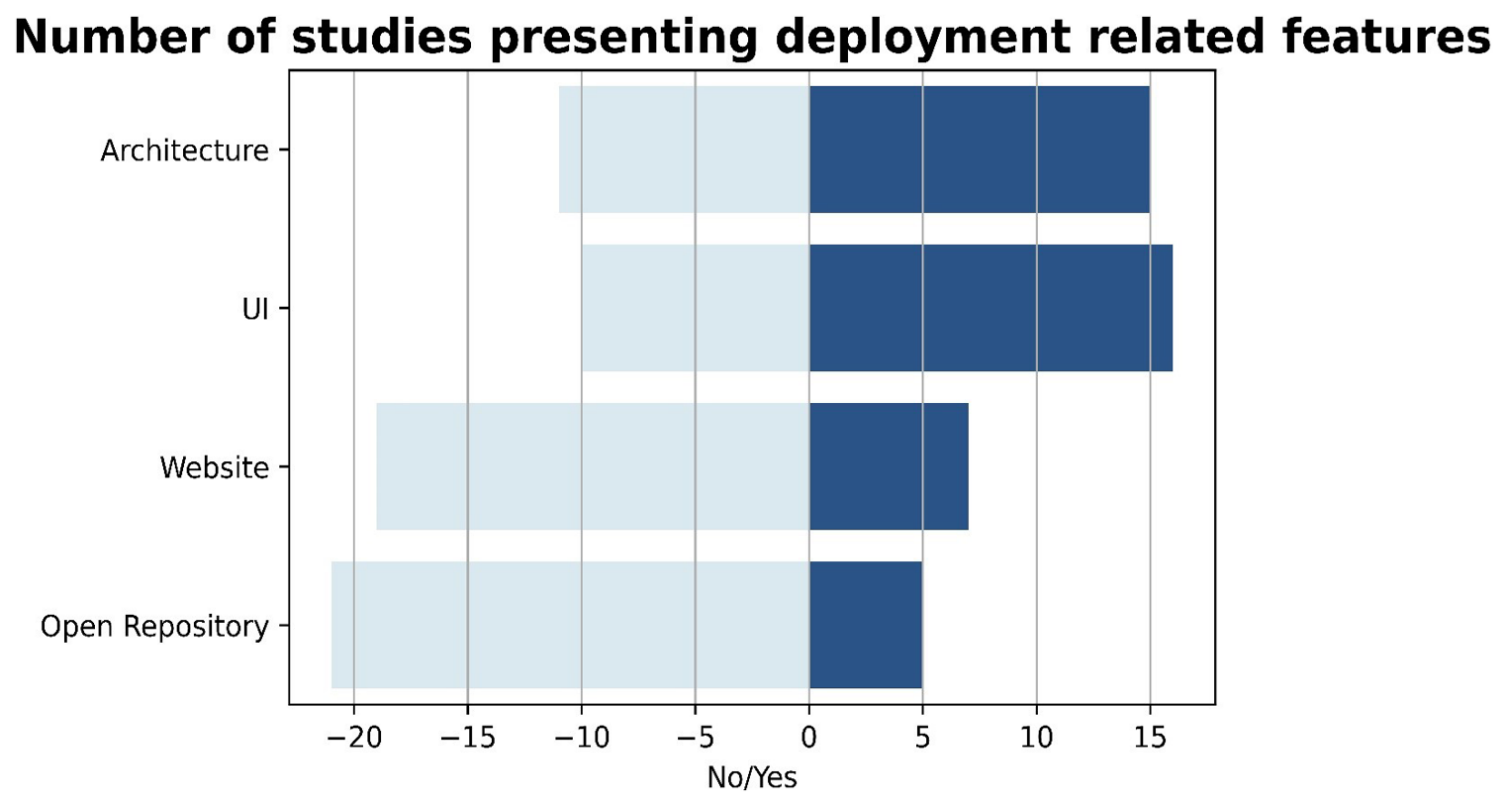
Chart presenting the number of studies that provided information of deployment related features.

All of the aforementioned results are visualized in Figure 7, to better facilitate the reader in understanding the aggregated results.

***4.3.2. Classification and Analysis of most prominent Software Technologies.*** The second layer of the technical characteristics analysis refers to the classification of the various technologies used for the marketplaces development and presented in the corresponding primary studies. It is evident that in several studies, not all or any of the technologies were showcased. Hence, the purpose of this analysis is to highlight the most prominent technologies used, allowing for the better understanding of the digital marketplace ecosystem in the field of EU-funded projects and for facilitating future research based on the State-of-the-Art technologies. Furthermore, since a significant number of papers (8 out of 26 studies) utilize blockchain technology, the analysis is divided into two parts: the first focuses on presenting the aggregated results gathered regarding the technologies used, while the second focuses only on the blockchain-related technologies and is thoroughly presented in Section 4.3.3. Overall, the technical analysis aims to provide a more focused and comprehensive examination of the technologies used in digital marketplaces.

The identified technologies were grouped into four broader categories:

(1) **Frameworks** ( Edwin, 2014): A framework typically includes a set of standardized practices, concepts, rules, conventions, and best practices that guide the development process.
(2) **Computer Languages** ( Lando *et al.*, 2007): Formal languages used to communicate instructions to a computer. Several types of languages exist that serve different purposes. For instance, programming languages are used to create software applications and programs, while markup languages are used to store and transport data.
(3) **Data & Storage technologies** ( Carullo *et al.*, 2017; Siddiqa *et al.*, 2017): Data and Storage technologies refer to the technologies and techniques used for managing and storing data, information, and objects in computer systems.
(4) **Communication technologies** ( Lascano & Clyde, 2016): Communication technologies refer to several sets of protocols, rules, standards, and objects that enable two or more entities to exchange information and facilitate transfer of data or information between different systems or platforms, which may use different communication protocols or data formats.

**Figure 8.**
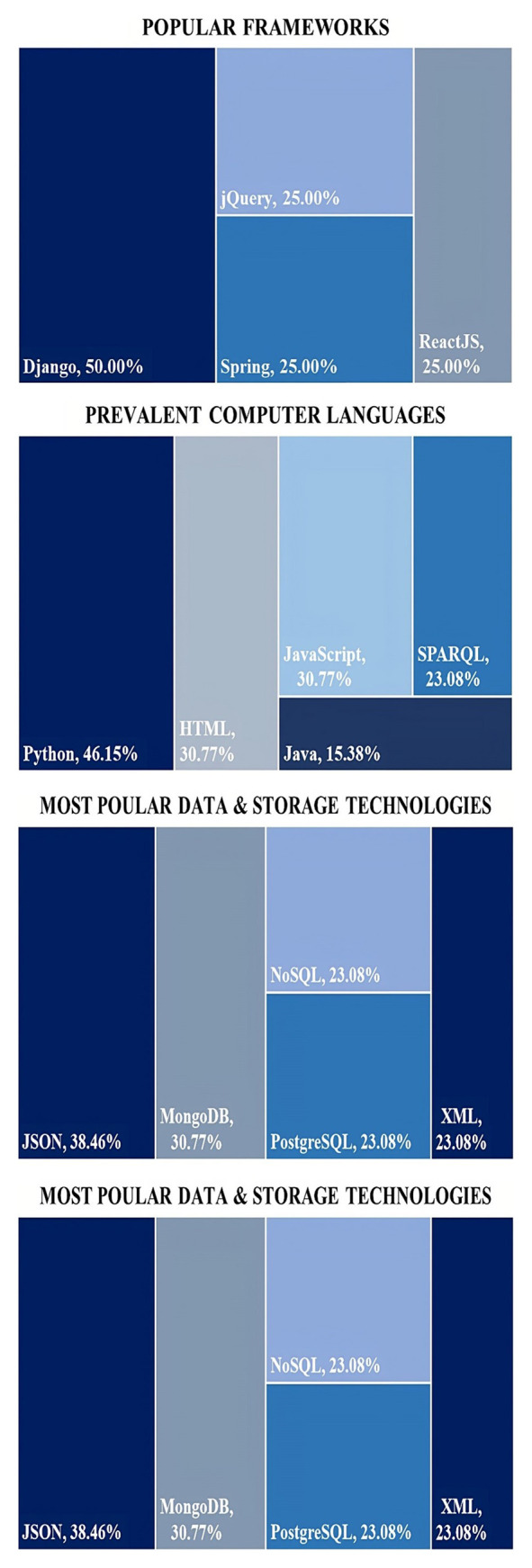
Multiple treemap charts depicting the most prevalent technologies used across the primary studies.

Table 7 provides a summary of the technologies employed in each primary study, offering a comprehensive overview of the digital marketplace landscape across the literature reviewed. Additionally, the technologies that were most frequently utilized in the primary studies for each category are showcased in Figure 8. It should be noted that the frequency percentages presented for each category were based solely on the studies that provided information on technologies within that category and did not include those that did not.

**Table 7.** Classification of technologies.

No.	Frameworks	Computer Languages	Data & Storage technologies	Communication technologies
a1	Assert4Soa	SerDiQueL	-	-
a2	Django; jQuery	Python; SASS; HTML	-	-
a3	-	C#; C++; Java; JavaScript	-	HTTP
a4	-	SPARQL	-	REST APIs
a5	-	-	-	-
a6	Django	Python	JSON	-
a7	Django; Spring; ReactJS	Python; Bash; JavaScript	SQL; NoSQL; MinIO; Elasticsearch	REST APIs
a8	-	-	-	-
a9	-	-	-	-
a10	Spring; Hibernate ORM	SPARQL; Java	MySQL; SQL; MongoDB; PostgreSQL; NoSQL; GraphDB Free	-
a11	-	-	JSON	-
a12	-	-	MongoDB; XML; Open Virtualization Format	-
a13	-	JavaScript	JSON	Kafka stream; HTTP; GraphQL
a14	-	Python	-	FED4FIRE API
a15	-	-	HDF5; RDF	-
a16	Bootstrap; SASL; jQuery	HTML; JavaScript	PostgreSQL; IPFS	LDAP; HTTP
a17	-	-	-	REST APIs
a18	-	-	-	-
a19	-	-	YAML	HTTP
a20	-	-	DRAM	OpenFlow; HTTP
a21	-	-	MongoDB	Delayed Message Queue
a22	-	-	XML	-
a23	Django; ReactJS; Angular	HTML; Python; Typescript	PostgreSQL; MariaDB; XML; Elasticsearch; JSON	REST APIs; HTTP
a24	-	SPARQL; RDF Query Language	RDF	-
a25	-	-	-	-
a26	Flask	Python; CSS; HTML; PHP	MongoDB; NGINX; Gunicorn; NoSQL; JSON	REST APIs; HTTP

In the analysis of the primary studies, it was found that 50% of them utilized the Django web framework, making it the most commonly used framework. Django is a high-level Python web framework that enables the efficient and practical development of web applications. For example, Rehm *et al.* (2021) utilized Django to develop their web application, showcasing the capabilities and effectiveness of the framework in web development. The next most common frameworks used in the primary studies were jQuery, Spring, and ReactJS, each with a frequency of 25%. jQuery is a fast, small, and feature-rich JavaScript library that simplifies HTML document traversal and manipulation, and event handling, which was utilized by Palaiokrassas *et al.* (2021). Spring is a popular Java-based framework that provides a comprehensive programming and configuration model for modern Java-based enterprise applications ( Bassiliades *et al.*, 2018). Lastly, ReactJS is an open-source JavaScript library used for building user interfaces or UI components, utilized by Rehm *et al.* (2021).

The next category evaluated for this technical analysis refers to the most common computer languages observed among the primary studies. The broader term “Computer Languages” was used to encompass all different types of languages (i.e., programming, markup, query, etc.) used in the primary studies both for front-end and back-end development. The analysis of computer languages revealed that Python was the most prevalent language, with 46.15% of the studies utilizing it and this is quite anticipated also from the framework analysis. Python is a versatile language and is often used for full-stack web development, as described by Stavropoulos *et al.* (2020), or for executing certain services via REST API calls, as Rehm *et al.* (2021) mention. JavaScript along with HTML followed next with 30.77% of the studies utilizing them. JavaScript is primarily used for client-side web development, although it can also be utilized to handle the interaction with smart contracts ( Palaiokrassas *et al.*, 2021), while HTML is a commonly used standard markup language for creating web pages ( Rehm *et al.*, 2021). The next most common language was SPARQL which was utilized in 23.08% of the primary studies. SPARQL is a query language for handling RDF data, and was utilized accordingly by Bavota *et al.* (2014). Lastly, Java was used in 15.38% of the primary studies. Java is a popular general-purpose programming language often used for enterprise applications, utilized by Bassiliades *et al.* (2018). Overall, these findings suggest that Python, JavaScript and HTML are the most commonly used languages for developing applications related to the technologies covered in this systematic literature review and these findings are also in accordance with well-known indexes such as TIOBE Index ^6^.

Data & Storage technologies were identified as a prominent category in the primary studies, with JSON being the most commonly used technology at 38.46%, followed by MongoDB at 30.77%. Other common technologies used include PostgreSQL, NoSQL and XML, all of which were utilized in 23.08% of the primary studies. JavaScript Object Notation (JSON), is a lightweight data interchange format that is easy for humans to read and write, while also being easy for machines to parse and generate, as Shi *et al.* (2022) describe. MongoDB and PostgreSQL are both popular database management systems, which can also be used in combination as presented by Bassiliades *et al.* (2018), with MongoDB being a NoSQL database, and PostgreSQL being a relational database management system. NoSQL (Not Only SQL) databases are used for storing and managing large volumes of unstructured or semi-structured data, and can handle flexible data models, provide horizontal scalability, and support high-performance data processing; thus, it was utilized accordingly by Mavrogiorgou *et al.* (2022). Lastly, XML, or Extensible Markup Language, is a markup language referenced by Bavota *et al.* (2014), as an example of its wide usage for storing and exchanging data on the web. The prevalence of these data and storage technologies highlights the importance of managing and storing data effectively in web-based applications.

The last category evaluated refers to Communication Technologies. The most common technology used in this category is HTTP at 58.33%. This is not surprising since HTTP is a fundamental protocol for data communication on the World Wide Web, which offers platform-independence and interoperability, and was utilized accordingly by Westera *et al.* (2019). RESTful APIs are the next most common communication technology used in the primary studies, with a percentage of 41.67%. As Pincheira, Vecchio, and Giaffreda (2020) describe, RESTful APIs have gained popularity due to their ability to offer a standardized approach for creating web services that enable diverse components to communicate with each other seamlessly, making them compatible with various client applications. HTTP and REST APIs are the most prevalent communication technologies due to their ability to provide a standardized, flexible, and efficient way for systems to communicate and to meet different needs.

**Figure 9.**
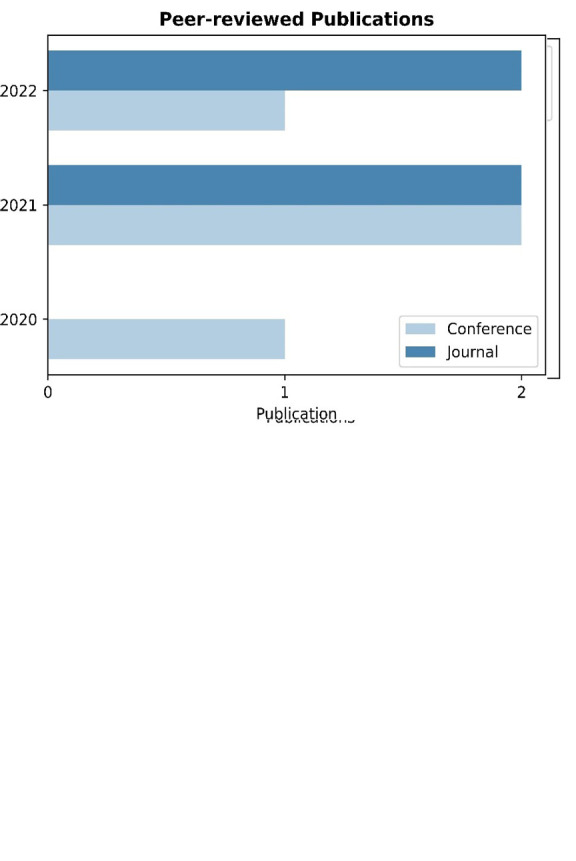
Blockchain-related papers distributions.

***4.3.3. Blockchain-related analysis.*** The last layer of the technical analysis pertains to the presentation of the blockchain-specific details gathered and examined from blockchain-based papers (as showcased in Figure 9). The papers relevant to blockchain are numbered to be eight, with the first one published in 2020 and the majority published in 2021. The works in the literature are related to different marketplace types, with the majority of them focusing on data (50%), reflecting the emergence of data marketplaces after 2018 (as analyzed in Section 4.1). Data marketplaces use blockchain to allow users to have control and incentives for sharing their data, by promoting disintermediation and democratization in data exchange. Similarly to data, there are suggestions in services (37.5%) and marketplaces with a wide variety of products (12.5%). The products’ complexity grows as the community gets accustomed to the blockchain. Conferences and journals accommodate the publications with an equal split (each source has 4 related publications).

The aforementioned papers use a range of technologies for delivering a functional marketplace for end-users. Blockchain research divides the concepts into four categories for analysis based on the considerations of the blockchain features and user requirements for an intuitive DApp:

(1) Infrastructure
(2) Smart contract development
(3) Technologies for deploying a DApp
(4) Testing and debugging tools

Blockchain merges hardware and software with cryptography to establish a decentralized network where nodes can securely exchange information. Initially, the analysis commences with the review of the blockchain protocols, which provides a level of abstraction on the technology stack executed by the nodes. The applications suggested in the literature benefit from various blockchains varying in the network’s type and permissions. Ethereum ^7^ is the first blockchain to implement a Turing-complete machine for executing smart contracts. As a result, it is the favored out of the public permissionless networks indicated by the concepts established on Ethereum ( Fotiou *et al.*, 2021; Niya *et al.*, 2021; Pincheira *et al.*, 2020). On the other hand, some concepts rely on private permissioned blockchains using frameworks to operate their networks, like Corda R3 ( Fernández-Fernández *et al.*, 2021), Quorum ( Palaiokrassas *et al.*, 2021), and Hyperledger Fabric ^8^. Finally, an intriguing use case is the selection of Alastria ( Palaiokrassas *et al.*, 2021), which is a public-permissioned platform.

The introduction of smart contracts is vital for the distributed ledger technology as triggers the transition to the second generation of the blockchain. Essentially, smart contracts are the encoded logic of procedures executed in a decentralized manner by the network’s nodes. While developers can opt for new smart contract languages like Solidity ( Palaiokrassas *et al.*, 2021), there are frameworks in other languages like JavaScript ( Genés-Durán *et al.*, 2022) and Python’s web3 ( Pincheira *et al.*, 2020) for deploying smart contracts.

Despite blockchain being an integral component for developing a decentralized application, there is a need to implement other components for the completion. There are suggestions discussing the security and identity aspect of the marketplaces by implementing standards like OpenID and OAuth2 ( Palaiokrassas *et al.*, 2021) and frameworks like Keycloak ( Palaiokrassas *et al.*, 2021) to apply these standards. In the case of authentication based on blockchain, the identity management has been facilitated by the JavaScript’s Web3 library ( Niya *et al.*, 2021) in order to utilize Ethereum addresses. Finally, the literature refers to developing and testing smart contracts in a lab environment. Most of the tools used do not require their deployment on public networks but still provide metrics like gas and associated fees, and Frameworks for development and testing are mainly Ganache ( Palaiokrassas *et al.*, 2021; Pincheira *et al.*, 2020) and Remix ( Palaiokrassas *et al.*, 2021).

***4.3.4. Rest of technical-related analysis.*** This Section delves into further technical-related analyses beyond deployment features, software technologies, and blockchain specifics, touching on areas vital for enhancing marketplace functionality and user experience. It is essential to mention that, although a similar detailed and aggregated analysis as conducted in previous subsections was not feasible for following technical aspects due to the limited number of studies directly addressing them, these elements have been included due to their importance in the development and operation of digital marketplaces. The topics that are analyzed in the following paragraphs play pivotal roles in the secure, efficient, and user-friendly operation of digital marketplaces.

**OAuth**

In the context of digital marketplaces, OAuth has emerged as a powerful tool and is often the first step in the process of accessing a web platform (or marketplace in our case), providing both authorization and authentication to ensure secure and seamless integration between multiple platforms and applications. While only a few papers provided details on how they handle authentication and authorization processes, the topic was still explored in primary studies. OAuth is particularly relevant for digital marketplaces that rely on external APIs to access user data ( Stavropoulos *et al.*, 2020), as it provides a secure and standardized way to delegate access to these resources. OAuth can also be used in conjunction with other technologies, such as JSON Web Tokens (JWTs), to securely transmit information between parties, further enhancing the security and usability of OAuth in digital marketplaces ( Rehm *et al.*, 2021). In addition, by allowing users to grant controlled access to their protected resources, OAuth provides a standardized framework for digital marketplaces to share access and functionality in a way that benefits all parties involved ( Palaiokrassas *et al.*, 2021). For digital marketplaces, the use of OAuth can offer several benefits, including increased interoperability, improved user experience, and enhanced security. By leveraging the power of OAuth, these marketplaces can enable seamless data exchange between different systems and applications, facilitating the creation of a vibrant and interconnected digital marketplace ecosystem.

**Ontologies**

Ontologies were identified as a relevant aspect in this SLR, however only a limited number of studies provided information about them, making it difficult to conduct an aggregated analysis. Despite the limitations, the topic of ontologies was still explored in the primary studies, and it is worth noting that their absence in some domains may be attributed to their resource-intensive nature and that they require significant expertise, which may lead to difficulties in their use. Digital marketplaces are evolving to become increasingly complex, considering the diverse range of products and users they accommodate. This new paradigm allows for the inclusion of both tangible products such as sensors ( Stavropoulos *et al.*, 2020), as well as intangible products such as software applications. Users set their requirements and desire to query the products based on their individual features ( Ali *et al.*, 2013). The marketplaces’ complexity increase as the target audience can be global and have different backgrounds. For example, healthcare marketplaces ( Stavropoulos *et al.*, 2020) accommodate services and products for developers and other stakeholders.

Ontologies are deployed in the marketplaces to address the above issues, as they enhance the interconnection and openness of the marketplaces’ resources and ultimately result in their findability, and also provide a structured way to represent knowledge. For instance, Bassiliades *et al.* (2018) utilize the DUL upper level ontology, a domain-independent ontology, to enhance semantic interoperability with similar projects and research endeavors, favoring extensibility since adding new classes and properties is made easier. One of the most known ontologies is OWL, utilized by Horsch *et al.* (2020), to better define concepts, relationships, and properties in a structured and systematic manner, enabling thus greater interoperability and reusability of data. Finally, ontologies can also help users link and monetize their resources like data marketplaces ( Roman *et al.*, 2017).

**Data & Storage**

As already presented in Section 4.3.2, the primary studies reviewed detail the text file formats for facilitating the storage and exchange of data between applications and systems, with JSON being the most popular. However, there are similar technologies found across the literature that facilitate different needs. For example, YAML is a human-readable data serialization format that is often used for configuration files ( Bernardinetti *et al.*, 2021) and data exchange between programming languages.

The data format is essential for standardizing the format for facilitating communications and reducing errors’ likelihood, while the file formats relate to the underlying storage infrastructure permitting information to be stored and transmitted. The storage infrastructure requirements differ in each work, as the stored data can be structured and unstructured. For example, there are contemporary deployed platforms that utilized relational databases, but there are also works in the literature with the more flexible approach provided by NoSQL platforms. However, a combination of both technologies is observed across the literature ( Bassiliades *et al.*, 2018; Faliagka *et al.*, 2022). The combination of SQL and NoSQL databases is often referred to as NewSQL or hybrid databases, where the benefits of both SQL and NoSQL databases are combined to achieve improved scalability, performance, and flexibility. In this approach, SQL databases are typically used for transactional processing and complex queries, while NoSQL databases are used for high-speed data ingestion, processing, and retrieval. The two types of databases can be integrated using a variety of approaches, including data replication, data synchronization, and data sharding.

Furthermore, there are decentralized systems implemented in the literature for storing data, like IPFS ( Palaiokrassas *et al.*, 2021), which is a protocol and network designed to create a distributed file system that allows users to access and store files in a decentralized manner. Finally, storage systems should be considered alongside blockchain implementations. The aforementioned platforms are viable as off-chain storage, while blockchain can store transactional data.

**Containerization**

Containerization is a method that is frequently used in the process of software deployment. This method bundles an application’s source code, files, and libraries so that it can run on any infrastructure ( Watada *et al.*, 2019). In terms of marketplace deployment, containerization can have two distinct applications covered in the literature. The marketplace is a software of different components interconnected to each via their logical architecture. All these components composing the marketplace can use containerization to simplify the deployment. The second use case for containerization is associated with the distribution of applications to be utilized in the market. There are marketplaces encouraging developers to publish their applications. Containerization is basically the standard that marketplaces provide to developers to follow when they publish their applications and ensure the execution on the user’s infrastructure.

The two most commonly used technologies that were found in the primary studies of this SLR are Kubernetes and Docker. Docker is a platform for building, packaging, and distributing containerized applications ( Rehm *et al.*, 2021), while Kubernetes is an orchestration system for automating the deployment, scaling, and management of containerized applications across a cluster of nodes ( Faliagka *et al.*, 2022). Hence, Docker provides the tools to create and manage containers ( Faliagka *et al.*, 2022), while Kubernetes provides the tools to manage and orchestrate multiple containers across multiple hosts ( Niya *et al.*, 2021). Continuous integration ( Rehm *et al.*, 2021) is an important aspect of software engineering that can be facilitated through the use of deployment software such as Jenkins and Kubernetes’ Helm ( Rehm *et al.*, 2021). When combined with containerization, these tools can help to further streamline the deployment process and improve the overall efficiency of marketplace deployment.

As the marketplace relies on different components, there is a need to document the changes and details for all the deployed ones. For this reason, literature papers include tools for aiding developers in versioning their code, as analyzed in the following subsection.

**Collaboration tools**

The use of collaboration tools in software development has been found to facilitate teamwork and increase productivity. However, it is important to note that not all projects or teams may find collaboration tools suitable or effective, especially those that require strict access controls, have concerns about intellectual property, or have difficulties with version control and coordination among team members.

The suggested review found that the most commonly used tools found across the literature, are GitHub ( Palaiokrassas *et al.*, 2021) and GitLab ( Faliagka *et al.*, 2022; Rehm *et al.*, 2021). These platforms provide a centralized location for developers to work together on code, track changes, and manage the development process, and through their use, teams can streamline their software development process and increase productivity. Finally, another tool found across the literature was Figma ( Alvsvåg *et al.*, 2022), which is a design and prototyping tool that enables teams to collaborate on user interface (UI) and user experience (UX) design, and also provides features such as real-time collaboration, commenting, and version history.

### 4.4. Research-related Results

This section investigates the core research aspects of the studies included in our systematic literature review, aiming to provide a holistic view of the research dynamics within the domain of digital marketplaces fostered by EU funding. For a better overview of the findings, a summary table is presented below ( Table 8), where the scope diversity of the research studies is obvious: from the presentation of complete and holistic marketplaces in various industry sectors to more methodology focused research studies applied to a marketplace.

**Table 8.** Major Research Outcomes.

No.	Major research outcomes
a1	This study introduces a marketplace prototype that leverages the Assert4Soa framework to offer business software with certified security properties. It aims to mitigate security concerns in cloud adoption by providing a transparent platform where users can compare software based on security certifications. This solution helps businesses comply with security standards and regulatory requirements, facilitating informed procurement decisions for cloud services and applications.
a2	The ACTIVAGE marketplace uses a hybrid logic- and text-based method for discovering Active and Healthy Ageing IoT applications. The authors employed semantic analysis to enable seamless discovery and deployment of AHA solutions. By integrating blockchain for security, and smart contracts for transparency, it addresses key challenges in deploying IoT applications for ageing populations, promoting a unified portal to enhance accessibility and innovation in the sector.
a3	The RAGE project introduces a marketplace to initiate innovation in serious gaming by offering a central platform for sharing game technologies and resources. Aimed at connecting researchers, developers, and other stakeholders, it facilitates the dissemination and application of game technology components. With an initial offering tested through seven serious games, it invites contributions, aiming to be a technology-neutral hub that enhances innovation potential.
a4	The proDataMarket marketplace aims at facilitating the publication, discovery, and monetization of linked data, with a particular focus on geospatial linked data. This data marketplace enables data providers to monetize their data while offering consumers simplified access to valuable geospatial insights. Through demonstration and architectural design, it showcases how linked data can support diverse domains beyond its initial geospatial focus, promoting flexibility, utility, and innovation in data usage and sharing across various sectors.
a5	The study introduces the first European marketplace for trading used F-gases, aligning with EU's F-gas Regulation and the Green Deal to support the circular economy of refrigerants. It facilitates the trade of recovered F-gases, ensuring transparency and secure transactions. The marketplace, currently operational in Slovakia, Hungary, Czech Republic, aims for wider European expansion. It features user verification, wallet management, and SEPA transactions.
a6	The study presents a decentralized blockchain-based marketplace for Fog/Edge computing resources. Through experimental setup and live network analysis, it evaluates the feasibility, cost, and performance of blockchain technology for creating a marketplace that ensures transparency, security, and trust. The findings demonstrate the potential to facilitate a decentralized infrastructure for trading computing resources, and also contribute to the understanding of blockchain's applicability in enhancing the reliability of distributed computing environments.
a7	The SMART4ALL marketplace promotes collaboration in IoT and customized low energy computing, utilizing open-source technologies for flexibility. In its initial six months, it engaged 840 members from 44 countries and hosted 184 artefacts. It features a matchmaking service powered by AI, aligning user needs with available resources. It aims at expanding its network, enhancing semantic search, and integrating with other platforms.
a8	This study explores the creation of a data marketplace prototype to enhance data sharing, reuse, and collaboration in smart cities. By employing iterative prototyping and expert evaluation through semi-structured interviews, the authors demonstrate the potential of a data marketplace in facilitating innovative services and improving urban living. The findings indicate that such a marketplace is feasible and also valuable in promoting data-driven innovation and collaboration among city stakeholders, by also contributing to smarter and sustainable urban areas.
a9	This study presents the decentralized blockchain-based data marketplace i3-MARKET and DEFS, a free data sampling service, integrated into i3-MARKET. DEFS allows consumers to preview data samples before purchase, building trust and ensuring data quality. The service enhances marketplace efficiency by streamlining payment processes and resolving conflicts, aiming for broader applicability with future expansions to support both crypto and fiat transactions. This approach marks a significant step towards trustworthy and transparent data trading environments.
a10	The authors present a Platform-as-a-Service marketplace that avoids provider lock-in by using a semantic model based on DUL ontology. Through systematic reviews of Cloud platforms and leveraging standards like CAMP and TOSCA, they developed a flexible, extensible ontology for annotating PaaS offerings and application requirements. It supports efficient matchmaking between appls and Cloud PaaS offerings, addressing interoperability and portability challenges.
a11	The authors present a novel approach to building trustworthy cloud marketplaces through blockchain technology. By integrating auction-based service selection and decentralized witness mechanisms within SLA management, they present a transparent, fair, and inclusive marketplace environment. Utilizing Ethereum, AWESOME demonstrates practical viability through extensive evaluations on execution latency and cost, revealing its economic and operational feasibility.
a12	The authors introduce a marketplace, offering a seamless environment for trading cloud-based services. Employing a service- oriented architecture, it facilitates the modular composition of services, supports advanced pricing models, and incorporates BI for enhanced service discovery, selection, and trading. Evaluation through expert feedback and real-world scenarios demonstrates its potential to transform cloud service trading, which benefits both providers and consumers.
a13	ITrade is a blockchain-based marketplace for IoT data streams, focusing on decentralization, scalability, and data sovereignty. Employing Smart Contracts, it facilitates secure data exchange between IoT device owners and data buyers. Its architecture enhances user experience by simplifying deployment and reducing costs, with evaluations confirming its efficacy and compliance with data sovereignty principles.
a14	This study introduces a marketplace to enable cloud network slicing across multiple domains, addressing critical aspects of 5G networking. The NECOS platform facilitates dynamic discovery and allocation of slice resources. It is backed by an architecture and information model that supports the dynamic creation of end-to-end network slices, leveraging resources from various infrastructure providers. Its model is demonstrated through an initial implementation, showcasing its potential to enhance resource utilization and flexibility in network slice provisioning.
a15	The authors develop an open marketplace platform for materials modeling services, utilizing ontologies for service characterization, user interactions, and data management. The ontologies, facilitate the annotation, ingest, and retrieval of data, supporting semantic interoperability within and across modeling platforms. The marketplace aims to cater various users by offering diverse services, such as training, model parameters, and simulation platforms, with the goal of enhancing accessibility and usability of modeling and simulation in thermodynamics and mechanics.
a16	This study showcases a system that integrates blockchain technology, smart contracts, and IoT to create a secure, decentralized data marketplace for smart cities. The marketplace enables stakeholders to trade sensor data and services securely and anonymously, supported by a custom token for transactions. Tested across cross-border trials, including Santander and Fujisawa, the system demonstrates interoperability, efficiency, and adherence to data protection principles.
a17	This paper presents a decentralized marketplace for multi-party collaboration in 5G networks, leveraging DLT to ensure secure, transparent trading of 5G resources across different domains. The marketplace facilitates dynamic service provisioning and resource sharing, enhancing trust, privacy, and distributed transactions. An initial proof-of-concept implementation demonstrates its feasibility for real-time resource trading in 5G ecosystems, emphasizing the potential of DLT to streamline operations and foster innovative services in next-generation networks.
a18	This study introduces a marketplace facilitating the trade of information between data providers and consumers, tailored for smart-grid data sharing. Leveraging local differential privacy and Ethereum, it ensures the privacy of data providers against consumers and system operators, by also enabling fair exchange and immutable transaction logs. It addresses the critical balance between privacy protection and the utility of shared data in generating meaningful statistics.
a19	Nautilus revolutionizes cybersecurity training by automating the deployment of training scenarios and enabling a marketplace for sharing vulnerabilities and scenarios. It simplifies creating and managing cyber ranges with cloud technology and a user- friendly interface, promoting community collaboration. Early feedback from usability tests reflects its potential to enhance cybersecurity education and knowledge exchange.
a20	FORTIKA introduces a cybersecurity solution for SMEs, integrating hardware and software within existing network gateways. It guides SMEs to trusted services via a marketplace, offering tailored cybersecurity solutions. Preliminary evaluations show the marketplace's efficiency in service deployment, demonstrating FORTIKA's potential to streamline cybersecurity for European SMEs with customizable, adaptive solutions.
a21	ECHO integrates diverse cyber range capabilities into a unified platform, enabling complex cybersecurity training and testing scenarios across sectors. It acts as a centralized portal for managing cyber training exercises, emphasizing scalability and the simulation of intricate environments. By combining various cyber ranges, it enhances training realism and effectiveness, advancing cybersecurity preparedness.
a22	This study introduces a blockchain-based TSO-DSO flexibility marketplace (EFLEX) that facilitates trading and flexibility services among TSOs, DSOs, and Prosumers. EFLEX provides a transparent, secure, and cost-effective way for managing energy flows and addressing congestion. Through blockchain technology and smart contracts, it ensures the secure and fair transaction of flexibility services. Pilot implementations in Bulgaria and Romania are highlighted, emphasizing in congestion management, TSO-DSO coordination, and marketplace dynamics.
a23	The ELG project aims to establish the primary platform and marketplace for Language Technologies (LT) in Europe. By offering an umbrella platform, it enables stakeholders to upload, share, and distribute LT services, products, and resources. Targeted to support all European languages, ELG plans to provide access to approximately 1,300 services and datasets, aiming to foster digital language equality and address the fragmentation in the European LT landscape.
a24	The MARKOS project develops a virtual marketplace for exploring open-source projects, emphasizing code structure, functionality, and licensing. It offers advanced browsing capabilities, inter-project dependency visualization, and legal issue identification. It supports sophisticated queries for better software reuse, addresses licensing compatibility, and promotes global access to open-source resources, making it a unique tool for developers and the open-source community.
a25	The study introduces a method to improve B2B marketplaces by seamlessly integrating purchasing and transport processes, leveraging semantic annotations and B2B standards. It offers a marketplace for product discovery, process integration, and synchronizing marketplace data with users' legacy systems. Validated in NIMBLE project, it enhances trading efficiency, reduces interaction efforts, and fosters trust through improved data interoperability and synchronization.
a26	The study introduces a data marketplace designed to offer an integrated platform for accessing and sharing various data assets, including AI/ML resources. Addressing the fragmentation of existing solutions, it provides structured, accessible data management tools and solutions, supporting both human and machine user’s data analysis and sharing needs. It aims to facilitate collaboration and efficient use of data assets, promoting innovation and knowledge sharing.

## 5. Discussion

**Figure 10.**
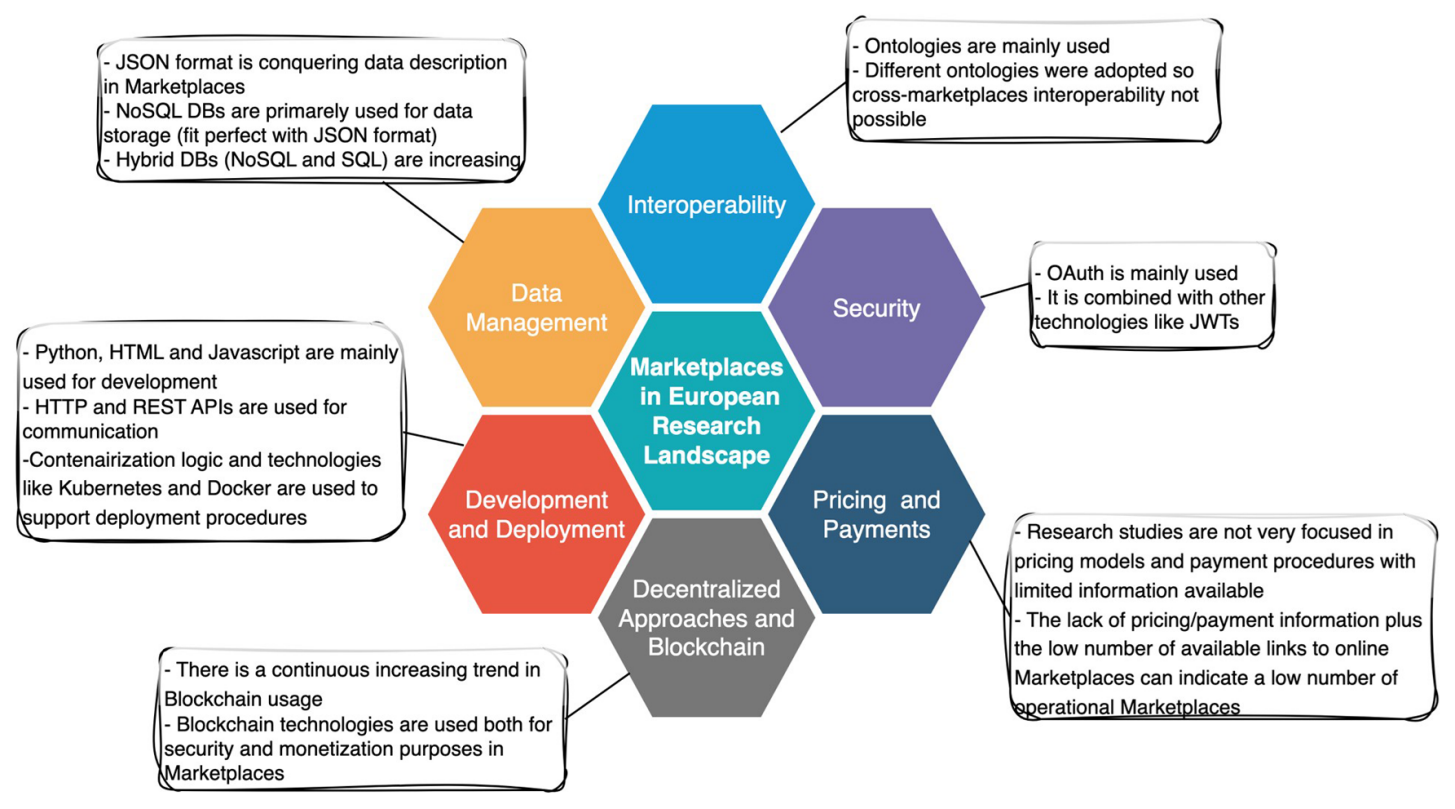
Highlights of SLRs Findings.

This Section provides a thorough discussion to offer deeper insight into the research challenges and opportunities that have emerged during the course of this SLR, with the identified issues being addressed in the following subsections. The main insights of current work as they are extracted from this discussion section and the previous analysis are depicted in Figure 10.

### 5.1. Discussion of Results

This study focused on digital marketplaces developed through European funding and in the course of European projects, which has not been specifically investigated in previous research studies. It also highlighted the significant potential of digital marketplaces as a means to enhance competitiveness and innovation in various industries and to stimulate novel research and industry sectors in the European region, since they offer various advantages, such as increased accessibility, convenience, and personalized recommendations.

Within these subject areas, this research work presents a technical classification system that encompasses general, business-related, technical characteristics and research-related results observed between the years 2013–2023, which could be used as a valuable tool for benchmarking and comparing different digital marketplaces based on their characteristics and as a framework for conducting market research and trend analysis in the digital marketplace industry. From the present SLR, 26 papers were examined and analyzed, from which specific data features were gathered and were thoroughly presented in Section 4. This rigorous analysis offered valuable insights and discovered trends in the digital marketplace ecosystem. Firstly, services, data, and multi-typed marketplaces were found to be the most prominent ones, probably attributed to the increased demand for digital services and the rise of big data, while multi-typed marketplaces by providing a diverse range of market offerings (including digital services, data analytics, and innovative product combinations), can meet various customer needs. In addition, the IoT is a developing industry, among the most common for these marketplaces, and their popularity can be justified by their ability to provide a convenient and efficient way for businesses to access and manage IoT data and services. Secondly, the combination of Django and Python was particularly prevalent in the studies analyzed, with many papers mentioning their ease of use and ability to quickly create web applications as a major advantage, while Python’s versatility and large number of libraries (including AI-related among others) and frameworks renders it as an attractive choice for digital marketplace development. In terms of data management, JSON (JavaScript Object Notation) was identified as a widely used technology due to its lightweight and easy-to-parse format, and its simplicity and compatibility with many programming languages. Lastly, HTTP and REST APIs were found to be the most commonly used communication technologies, due to their ability to provide consistent communication between different systems and devices, thus handling a large volume of requests. Finally, blockchain technology emerged as a prominent trend in digital marketplaces, offering benefits such as increased transparency, security, and trust. Its decentralized nature makes transactions more direct, since no central authority is required, while the immutability of blockchain-based transactions can enhance the overall trust and credibility in the marketplace, since all records are tamper-proof and can be verified by all parties involved.

### 5.2. Limitations of the study

While this study offers valuable insights into digital marketplaces developed through European funding and highlights their potential, limitations worth documented emerged throughout the process. Since only EU-funded projects’ publications with prototypes or fully developed marketplaces were considered, the publications’ number for analysis was capped by the criteria and excluded conceptual frameworks. Acknowledging that the criteria strictness can affect the extent of the digital marketplace landscape analysis, the snowballing process was carried out as a mitigation action to this threat ( Wohlin, 2014), considering all references listed in the selected papers. Secondly, the researchers’ technical analysis solely details the novel aspects of the components while disregarding information on the other functional components existing in the marketplaces (i.e., back-end processes, authentication, data storage and management, etc.). The information absence limits the ability to conduct an aggregated and comparative analysis of the overall and diverse marketplaces’ components. This could impact the usefulness of the technical classification system developed in this study for benchmarking and comparing different digital marketplaces, but to mitigate this risk, this topic was addressed in the Subsection 4.3.4 in order to provide a more nuanced and contextualized interpretation of our results. Lastly, business-related characteristics (revenue model, pricing strategies) are underrepresented in the technical description, restricting the comprehension of the digital marketplaces’ potential impact on various industries. A rigorous analysis was conducted as a mitigation to the aforementioned risk in the previous Subsection 4.2.1 to highlight the potential impact of business-related characteristics on the success of digital marketplaces.

### 5.3. Future directions

Based on the limitations and insights of this study, future researchers could consider exploring conceptual ideas for digital marketplaces to broaden the analysis of the landscape of marketplaces developed through European funding. Furthermore, more information is needed in regard to revenue models and pricing strategies, which could assist future studies examining the potential impact of digital marketplaces on various sectors of the economy. In addition, there is a need for more information on specific technical characteristics such as software and hardware components, system architecture and design, data storage and management, data security and privacy, performance metrics and scalability. A framework could be designed for future researchers to systematically collect and analyze relevant data, which could outline methods for benchmarking and comparing these features, in order to identify best practices and areas for improvement in marketplace development. Moreover, as blockchain technology emerges as a prominent trend, researchers could explore its impact on the development and success of digital marketplaces, also considering novel characteristics. For example, in some more recent approaches ( Nizamis *et al.*, 2024), Non-Fungible Tokens (NFTs) are also used for exchanging AI models over a digital marketplace. However, it is crucial also to investigate the potential ethical and legal implications of blockchain in electronic marketplaces, such as issues related to privacy, data security, and intellectual property rights. Finally, the results related to marketplace deployment clearly indicated that a lot of work should be done in the exploitation of EU-funded marketplaces in order to function smoothly after the project finalization and therefore, the community to benefit from their operation.

In addition to the above, the systematic literature review also raises important questions about the marketplaces’ societal implications. The observed trends have implications for policymakers and practitioners. This reflects a growing societal demand for greater control over personal data and the integrity of digital transactions, signaling a shift towards more decentralized and user-centric digital marketplaces.

Policymakers need to foster regulatory frameworks that support innovation while ensuring data protection, user privacy, and fair market practices. They should prioritize:

- Regulatory support for emerging technologies that enhance marketplace transparency and security, facilitating their adoption while safeguarding against potential misuse.
- Standards and guidelines for data sharing and transactions in digital marketplaces, ensuring they promote user trust and engagement.
- Encouragement of cross-sector collaborations to leverage digital marketplaces for societal benefits, such as improved healthcare access, environmental sustainability, and inclusive economic growth.

For practitioners, the emphasis on decentralized technologies and the integration of advanced data and analytics into marketplace operations suggests a strategic direction that aligns with evolving user expectations and regulatory landscapes. Practitioners should consider:

- Investing in user-centric design and development approaches that prioritize transparency, data control, and privacy as central features of digital marketplaces.
- Engaging with stakeholders across sectors to understand the broader societal impacts of marketplace technologies and to identify opportunities for creating shared value.
- Adopting agile and responsive strategies that enable quick adaptation to technological advancements and regulatory changes, ensuring sustained competitiveness and relevance in the digital economy.

In conclusion, the technological trends highlighted in this review call for a combined effort by policymakers and practitioners to steer the development of digital marketplaces towards positive societal outcomes. This involves not only accepting technological innovations but also addressing the ethical, legal, and social considerations they entail, ensuring that digital marketplaces contribute to a more transparent, secure, and inclusive digital future.

## 6. Conclusions

Digital marketplaces represent electronic platforms dedicated to the sale, distribution and marketing of various tangible and intangible “products” in the era of business digitalization, with software, data and services marketplaces being amongst the most popular. This study has systematically investigated the landscape of digital marketplaces delivered by EU funding, highlighting their diverse types, underlying technologies, deployment strategies, and their impact on various sectors. The conclusions drawn from this study underline the important role of EU-funded projects in advancing digital marketplace innovations and their contribution to addressing contemporary economic and societal challenges. However, the low number of scientific papers related to Marketplace coming from EU funding projects highlights that the developed Marketplaces in various EC projects are not documented in the scientific bibliography by the corresponding researchers and developers.

The analysis performed in this research study revealed a wide array of marketplace types, reflecting the adaptability of digital marketplaces to cater to different economic sectors and user needs, fostering a dynamic and versatile digital ecosystem within the EU. It also explored how technological advancements, and cutting-edge technologies foster secure and efficient digital platforms. Despite the successes, the study identified technological challenges and societal implications, which need to be addressed, since they are crucial for the sustainable growth of digital marketplaces.

## Ethics and consent

Ethical approval and consent were not required.

## Data Availability

No data associated with this article. *Repository name: Checklist for Digital marketplaces in European research landscape: A systematic literature review, https://doi.org/10.5281/zenodo.13869982* Data material for SLR, uploaded in Zenodo ( Nikoletos *et al.*, 2024) Creative Commons Zero v1.0 Universal.
