# Supplementary material for: Digital marketplaces in European research landscape: A systematic literature review
Source: Open Res Eur. 2024 Oct 17;4:223. [Version 1] doi: 10.12688/openreseurope.18657.1 (PMC11803373; doi:10.12688/openreseurope.18657.1)
Supplement: Peer review history

# Reviewer report: Reviewer response for version 1

**DOI:** 10.21956/openreseurope.20185.r47968

**Published:** 2024-12-17

**Authors:** Fadi Al-Turjman

**Affiliations:** 1 Near East University, Nicosia, Turkey

The rationale clearly stated its objective in the systematic review, laying emphasis on the advantages and application of the innovative technology in the digital marketplace in Europe by focusing on the objective landscape mapping I.e. within European Union countries, discussing about existing work and its application of the subject matter to identify gaps and discussing the future direction.

 Suggestions for Improvement:

 The author should make a brief explanation of the reason and present importance for the critical impact of this review with ongoing European Union initiatives, for example, (i) the relevance of the European science cloud for post-pandemic digital transformation in the marketplace (ii) the Horizon European program. (iii) The type of crypto wallet framework that can be recommend as best for cheap transaction gas fees. 

 The author has given a well-detailed explanation of the strategy they adopted for their research methods in various stages: the planning, conducting, and reporting stages, inclusion and exclusion criteria, and PRISMA

 LIMITATION

 1.    The article did not state how disagreement between selected articles was resolved.

 2.    The article doesn’t specify whether the reviewed articles underwent a peer review on a platform such as PROSPERO before execution.

 The author should consider using network analysis for collaboration study network

 Some parts of the conclusion are generalized without directly referencing the reviewed studies.

 The author should link some parts of the conclusion to specific findings in the review.

 The author should also use visual aids such as graphs and charts to summarize key findings such as gaps, trends, etc

Are the rationale for, and objectives of, the Systematic Review clearly stated?

Yes

Is the statistical analysis and its interpretation appropriate?

Partly

Are sufficient details of the methods and analysis provided to allow replication by others?

Partly

Are the conclusions drawn adequately supported by the results presented in the review?

Partly

Reviewer Expertise:

AI and IoT

I confirm that I have read this submission and believe that I have an appropriate level of expertise to confirm that it is of an acceptable scientific standard, however I have significant reservations, as outlined above.

# Reviewer report: Reviewer response for version 1

**DOI:** 10.21956/openreseurope.20185.r47458

**Published:** 2024-12-16

**Authors:** Mihaela Constantinescu

**Affiliations:** 1 Bucharest University of Economic Studies, Bucharest, Romania

This is a very useful research paper, as EU funded projects should draw the attention of academia and business alike, moreover if we talk about the creation of a marketplace platform, which will become the future of commerce. The authors have presented very carefully both the available literature, and their own research. Without diminishing the authors' credit for this work, I would make some remarks and comments, which actually don't interfere with my decision that this article should be indexed:

 1. In terms of research methodology, I have a comment related to the exclusion of paper from the grey literature. Although we can fully understand the academic perspective on grey literature, here the scope was to identify as much as possible the research conducted through European funds for marketplace projects, thus maybe a comparison between those researches presented in the grey literature and the other high-rated journals would have been very insightful, as the impact and sustainability represent two very important indicators for all European funded projects, and dissemination is included in these indicators.

 2. One question about the selection criteria: the authors have established that they will eliminate those papers that are focusing on a survey and those that don't present an implemented marketplace, but rather describe the concept or the idea of that digital marketplace. But what if the survey results led to the final concept of the marketplace, thus having there some relevant information about the European funded project results? Moreover, in papers published in high-rated journals, most of the times, you have to have some research results on which to base the whole paper. All these led to one that I would like to ask authors: why just those articles about already implemented marketplaces?

 I would say that for this reason of questions arising in relation to the selection criteria, maybe it would be better if the authors explain in short the reason for each criteria used in selecting the papers.

 3. In the results section, I would mention the following aspect: considering that the present paper is analysing the articles presenting marketplaces, it's not a marketplace products analysis, maybe the indicators about the marketplaces shouldn't be so many compared to the indicators about the articles. What I mean is that here the analysis is about those papers and how they are presenting work funded by European funds for marketplace platforms. Thus, from my point of view, it wound be more interesting to see a detailed analysis on section '4.4. Research-related Results', where each paper is evaluated in terms of its approach. Here it would have been interesting to see what type of research the authors of each paper have conducted and how.

Are the rationale for, and objectives of, the Systematic Review clearly stated?

Yes

Is the statistical analysis and its interpretation appropriate?

Yes

Are sufficient details of the methods and analysis provided to allow replication by others?

Yes

Are the conclusions drawn adequately supported by the results presented in the review?

Yes

Reviewer Expertise:

Secondary Data Analysis, Observation, Experiment, Survey

I confirm that I have read this submission and believe that I have an appropriate level of expertise to confirm that it is of an acceptable scientific standard.

# Reviewer report: Reviewer response for version 1

**DOI:** 10.21956/openreseurope.20185.r47452

**Published:** 2024-12-12

**Authors:** Santiago Andrés Azcoitia

**Affiliations:** 1 Universidad Politecnica de Madrid, Madrid, Spain

The paper provides a systematic literature review on EU-funded publications related to digital marketplaces. It lists those works, characterises the sample, and provides an analysis of business- and technical-related issues they deal with, identifying trends and issuing recommendations to policymakers and practitioners.

 I think the paper touches on a very relevant topic nowadays, as the number of related publications reflects, and is called to be relevant in the future, for example, to enable a market for data, software, models, and goods to make the most of digitalisation and AI. The methodology is well explained and I think the SLR can be reproduced in the future as needed. I think the scope is fine. My main concern is the structure, writing, format, and clarity of the paper. I will also provide my comments and suggestions for those more formal aspects of the paper. However, I will start by suggesting additional analysis in the paper that will contribute to increasing its quality and usefulness, in my opinion.

 Due to the limited number of studies in the scope, the sample is limited. Did the authors come across EU projects related to digital marketplaces that did not publish any paper on their architecture or design?

 I miss connections with the funding projects of those publications. Which are those projects? Can the authors include them in Table 4? They are usually referenced in the acknowledgement section of the papers. Also, the name of the marketplaces and links to their webpages, if available, would be useful.

 I also miss more information about the maturity achieved by those projects and marketplaces. This would be very interesting to evaluate the success of the funding actions. Has any marketplace become commercial? I think that the paper would include if it includes an analysis of the TRL the marketplace has achieved, as evaluated by the authors based on their investigation. Some features analysed in the paper, like the availability of a webpage, can be an input for this. Some more extra analysis will be needed, such as whether those websites contain active marketplaces, whether they link to existing real products, and how many users / providers and/or products they contain.

 Section 4.3.4 seems like random topics unrelated to the previous ones, but we discuss in storage SQL vs NoSQL technology, for which we have data in table 7. I would recommend the authors add whenever possible stats stemming from the SLR .

 In Section 5.2, I miss a key limitation of the study, which is the reliance in the information published by the projects/papers in the SLR. Also, in the conclusion the authors claim that a limited number of papers are available, this could also be considered as a limitation. Instead, the paper states some limitations and successful mitigation actions. If a potential limitation was sorted out somehow during the work, should it stay as a limitation in the paper?

 Section 5.3.3 includes some recommendations for policymakers and practitioners. However, I feel like some of them come out of the blue and are not necessarily connected with the SLR. For example, what in the SLR makes the authors recommend policymakers to prioritise regulatory support for emerging technologies, etc? Also, I agree that standards and guidelines are needed, but I would expect the analysis to point to the lack of standards as a challenge of data marketplaces and give some pointers to any standardisations if possible (e.g., in the ontologies, there is no standard to define data products, but some general-purpose initiatives like W3C DCAT or more niche like SDMX for statistical data are there. IDSA and Gaia-X projects are aiming at “standardising” data exchanges, not sure if there is any other initiative for other types of digital marketplaces). Bottom line, I think the authors must connect the recommendations with the findings of the analysis.

 In addition to these suggestions for improving the contents of the paper, my main concern is about the structure, writing, format, and clarity of the paper. Some recommendations follow.

 Regarding the structure, some recommendations and issues follow:

- Is Sect. 2.3 really needed?
- Section 3 can be split into planning, conducting and reporting subsections, and ordered properly. For example, inclusion criteria is more about planning but appear at the second half of this section.
- Table 3 can also relate the features to the questions of the study in section 3, and which of them are features to characterise the sample, which are technical and business related (like section 4).
- Section 4 does not introduce the following subsections
- Section 4.1 is more a characterisation of the SLR sample than “general results”, with general information about the selected studies.
- Table 4 can include the funding project, the name of the marketplace, and even citations of the papers as a measure of the impact. Some venues are acronyms, and other publication venues contain complete words.
- Besides, later in Section 4.3.3 the authors give again some “general results” of blockchain papers that I would move to Section 4.1. Also Figure 9 can be merged with global statistics of the sample as done in Fig 4, that separates blockchain and non-blockchain-based marketplaces.
- Also in 4.3.3 the authors enumerate four concepts in blockchain (infrastructure, smart contract, DApp tech, etc, but later in the text they do not use them in the classification. I would suggest following those four categories with four paragraphs highlighting the findings on each of them
- Long tables may be included in the appendix and leave just illustrative figures in the body of the paper. For example, I would move table 7 to the appendix, and leave figure 8 in the body of the paper.
- Section 4.4 is weird. After so verbose previous sections, this one is meaningless, just to introduce table 8. Is not table 8 a summary of the outcomes of the papers? I would move this table to the annex and use this section 4.4 to summarise key findings only, if any.
- In section 5.3. the authors go well beyond future research directions and give recommendations. I think this should be another section or, better, merged with the conclusions of the study (and shortened to avoid repeating ideas there).

Regarding the writing, I find the paper too verbose, repetitive (often going several times on the same idea), and difficult to read due to the length and lack of concision. There are many tables that contain useful information but distract the attention of the reader. The authors may consider moving them to an appendix, leaving just illustrative figures or summary tables in the body of the document. Moreover, a general proofread of the paper is needed to improve the language and make the paper more concise.

 The abstract does not summarise the paper thoroughly. For example, the authors give recommendations to practitioners and policymakers, which is not stated in the abstract. It could also be more specific on the trends and findings of the paper. My recommendation is a thorough rewriting of the abstract.

 The introduction could be cut significantly (e.g., is the 3 ^rd^ paragraph really needed?). I would also remove the following text: “For these reasons, the EU has encouraged the use of digital technologies to enhance competitiveness and innovation in the region, and digital marketplaces have been a key part of this effort. It is also worth mentioning that a main differentiation of the digital marketplaces from EU-funded projects in comparison with other marketplaces presented in the literature is that can also serve a higher community purpose, such as public health and security apart from exploitation purposes and is prominent to investigate if there are specific characteristics either business or technical toward this direction”. And proofread carefully, the abstract and the introduction must invite the reader to read the paper and writing can be significantly improved, in my opinion.

 Related works are also very verbose and can be significantly reduced. Also, I found Table 1 repetitive (maybe move it to an appendix?).

 In section 4.3 the authors introduce the subsections twice, first in the intro and then in each subsection 4.3.x. This is repetitive in my opinion.

 Regarding the format, I think figures can be made smaller and homogeneous in size and colour (Figure 8 seems to use a different palette of blues). Figure 1 format can be made more appealing. In figure 2, snowball adds one paper 25 -> 26, so I would say +1 paper (add the +) in the figure. In figure 5, why is the order of the industries like that? How do you come up with the list of industries? Any reference? This can be reduced or merged with Table 5 in a single table classifying the marketplaces (industry x type of marketplace ordered in descending order of importance, and number of marketplaces – with Table 5 in an annex. Figure 6 is weird with percentage and number of papers. Table 6 probably fits one column. In table 7, I would rather say “N/A” than “-“. Check typos in Figure 8 (POULAR -> POPULAR). Generally, I think most figures fit one column which will also contribute to shortening the paper. Finally, I would add the date of last access date to any hyperlinks in the footnotes, just in case they become inaccessible in the future.

 Regarding clarity, I think the paper must improve the connections between the different aspects discussed in the SLR:

- Research questions and features sought in the sample. For example, you tackle payments in the features but there is no question about payments
- Analysis and key findings that will be used later
- Recommendations and challenges/findings of the analysis that lead to them

Moreover, I think section 5.1 can emphasise more the findings and do it in a more concise way, as headlines and avoiding repeating again the analysis.

 Finally, some questions / random minor issues:

- In page 9 you state that “These results indicate that a significant proportion of the studies were more preliminary and exploratory in nature, as conference papers often feature early results or works in progress.”. In my experience, this depends on the discipline, and in some cases it is more difficult to publish papers in conferences.
- In page 14 you state that: “This suggests an increasing trend toward hybrid marketplace models in multiple economic sectors”. Maybe this can be supported with a figure showing the percentage of hybrid marketplace models or the mix of marketplace types over the years, as you do above with blockchain.
- In page 15 I would remove or relocate (and justify) the sentence about SEPA payments.
- In page 22 you speak about text file format to exchange data, like JSON, and then you talk separately about YAML, is not YAML text file format, as well?

I’d like to thank the authors for the paper. I hope this review helps improve it and makes it more useful for the community.

Are the rationale for, and objectives of, the Systematic Review clearly stated?

Yes

Is the statistical analysis and its interpretation appropriate?

Partly

Are sufficient details of the methods and analysis provided to allow replication by others?

Yes

Are the conclusions drawn adequately supported by the results presented in the review?

Partly

Reviewer Expertise:

Data economy, data marketplaces, machine learning, network economics, digital regulation

I confirm that I have read this submission and believe that I have an appropriate level of expertise to confirm that it is of an acceptable scientific standard, however I have significant reservations, as outlined above.
